# Best Practices for the Development, Scale-up, and Post-approval Change Control of IR and MR Dosage Forms in the Current Quality-by-Design Paradigm

**DOI:** 10.1208/s12249-014-0087-x

**Published:** 2014-03-01

**Authors:** Glenn A. Van Buskirk, Satish Asotra, Christopher Balducci, Prabir Basu, Gerald DiDonato, Angelica Dorantes, W. Mark Eickhoff, Tapash Ghosh, Mario A. González, Theresa Henry, Matthew Howard, Jason Kamm, Steven Laurenz, Ryan MacKenzie, Richard Mannion, Patrick K. Noonan, Terrance Ocheltree, Umesh Pai, Richard P. Poska, Michael L. Putnam, Ramani R. Raghavan, Colleen Ruegger, Eric Sánchez, Vinod P. Shah, Zezhi Jesse Shao, Russell Somma, Vijay Tammara, Avinash G. Thombre, Bruce Thompson, Robert J. Timko, Satyam Upadrashta, Sivakumar Vaithiyalingam

**Affiliations:** 1 Nonclinical Drug Development Consulting Services, LLC, Basking Ridge, New Jersey 07920 USA; 2 AHI Inc., Brampton, Ontario Canada L6W 4K4; 3 Novartis Pharmaceuticals, East Hanover, New Jersey 07936 USA; 4 National Institute of Pharmaceutical Technology and Engineering (NIPTE), Prospect, Illinois 60056 USA; 5 Bristol-Myers Squibb Co., Pennington, New Jersey 08534 USA; 6 Food & Drug Administration, Silver Spring, Maryland 20993 USA; 7 Merck & Co., West Point, Pennsylvania 19486 USA; 8 P’Kinetics International, Inc., Pembroke Pines, Florida 33027 USA; 9 Old Greenwich, Connecticut 06870 USA; 10 McNeil Consumer Healthcare, Ft. Washington, Pennsylvania USA; 11 Summit Pharma Solutions, LLC, South Windsor, Connecticut USA; 12 AbbVie, North Chicago, Illinois 60064 USA; 13 Janssen Research & Development, LLC, Spring House, Pennsylvania 19477 USA; 14 Purdue Pharma LP, Cranbury, New Jersey 08512 USA; 15 PK Noonan & Associates, LLC, Williamsburg, Virginia 23188 USA; 16 Sun Pharma USA, Cranbury, New Jersey 08512 USA; 17 Boehringer-Ingelheim VetMedica, Inc., Saint Joseph, Missouri 64506 USA; 18 Genentech, South San Francisco, California 94080 USA; 19 Janssen Ortho, LLC., Gurabo, 00778 Puerto Rico; 20 North Potomac, Maryland 20878 USA; 21 Arena Pharmaceuticals, San Diego, California 92121 USA; 22 Somma Tech Consulting, Somerset, New Jersey 08873 USA; 23 Nuron Biotech Inc., Exton, Pennsylvania 19341 USA; 24 Pfizer Inc., Groton, Connecticut 06340 USA; 25 AstraZeneca LP, Wilmington, Delaware 19850 USA; 26 Millennium Pharmaceuticals, Cambridge, Massachusetts 02139 USA; 27 Teva Pharmaceuticals, Pomona, New York 10970 USA

**Keywords:** CMC, ICH, IVIVC, PAT, QbD

## Abstract

In this whitepaper, the Manufacturing Technical Committee of the Product Quality Research Institute provides information on the common, best practices in use today in the development of high-quality chemistry, manufacturing and controls documentation. Important topics reviewed include International Conference on Harmonization, *in vitro*–*in vivo* correlation considerations, quality-by-design approaches, process analytical technologies and current scale-up, and process control and validation practices. It is the hope and intent that this whitepaper will engender expanded dialog on this important subject by the pharmaceutical industry and its regulatory bodies.

## INTRODUCTION

In 1991–1992, three scientific organizations—the American Association of Pharmaceutical Scientists, the Food & Drug Association (FDA), and the United States Pharmacopeia (USP)—collaborated to organize two workshops to explore the Scale-Up and Post-approval Change (SUPAC) principles for (1) immediate-release oral solid dosage forms (1991) and (2) oral extended-release dosage forms (1992). Proceedings from both workshops were published in 1993 (1,2) and have been used as guidance to the industry and regulatory bodies. The proceedings of both workshops discussed and defined the impact of (1) formulation or compositional changes, (2) process variable changes, (3) process scale changes, and (4) process site changes on the finished quality parameters of these products. Each area of change was further divided to reflect a hierarchy of “significance” and hence aided in establishing post-approval change filing documentation. In the case of the extended-release dosage forms, the potential need for the conduct of one or more pivotal bioavailability/bioequivalence (BA/BE) studies was recognized and, as a result, included a recommended decision tree to determine when a BE study would be needed to prove equivalence.

Although these documents continue to have utility in supporting post-approval changes, it has been recognized that there have been many improvements implemented in the scale-up and control of both immediate-release and extended-release oral solid dosage forms in the last two decades. It is the goal of the authors of this whitepaper to provide a concise updating of important development principles currently available to those in the industry involved in the development of such products. Accordingly, this whitepaper will present comprehensive chemistry, manufacturing, and controls (CMC) information to those involved in the development and review of oral solid dosage form dossiers. It is our hope that the added information will lead to improved process for post-approval changes. While much of this information is available in official guidance documents, for example, International Conference on Harmonization (ICH) and quality-by-design (QbD) documents, it is a goal of this whitepaper to bring all of the independent pieces into a unified document to facilitate improved understanding and implementation.

This whitepaper, sponsored by the Product Quality Research Institute, is a result of that thinking and is designed to engender additional discussion and commentary from experts within the industry, academia, and worldwide regulatory bodies. Although the document retains the spirit of the original workshop reports, it encourages the inclusion of new tools for the development, testing, and control of oral solid dosage forms. The use of tools and approaches such as process analytical technologies (PATs), QbD, *in vitro*–*in vivo* correlation (IVIVC), and more thorough excipient characterization should improve the robustness of the finished products and minimize or prevent unintended drift in the quality of the affected commercial drug products.

It should be noted that these best practices, as outlined in this paper, are only applicable to QbD-based applications. They cannot be used for legacy or mature products which were not developed using the new systematic and life cycle approaches. The principles of QbD are mainly in use in Canada, Europe, Japan, and the USA, but are gaining recognition in other countries as well.

## REVIEW OF THE 1993 ORAL SOLID DOSAGE FORM WORKSHOP REPORT FINDINGS

We begin our oral solid whitepaper with a brief review of the salient findings of the 1993 publications.

### Compositional Variables

In 1993, it was recognized that oral solid dosage forms contained both “noncritical” and “critical” components and that it was the job of the formulating scientist to establish (with data) which excipients fell into each category. Furthermore, it was recognized that for noncritical excipients, for both immediate- and extended-release (ER) products, “certain compositional adjustments (to formulations) were determined to be acceptable, without further justification” (2). The report also noted that “there is, however, an additional consideration for extended-release dosage forms: the inclusion of a critical release component(s) which enables the extended release of active ingredients. Thus, for extended-release dosage forms, consideration must be given as to whether the component is critical or not critical to drug release” (2). The passage of time has certainly confirmed the validity of these statements.

The authors have undertaken within this whitepaper to include a review of the current tools in place that allow pharmaceutical scientists and regulators to evaluate whether a change is critical or not critical.

Using tools in place today such as QbD approaches that explore and understand the relationships between target product parameters and end product quality attributes, our contributors believe that more robust products will result. We also feel that the use of the current improved statistical design packages when combined with QbD approaches can afford substantial information about the allowable range of both minor excipients/components and those that are critical to product performance. In addition, the use of these approaches when combined with improved testing techniques associated with PAT and enhanced finished product testing such as *in vitro* release can and should be used to facilitate review and approval of post-approval CMC submissions involving compositional variables. Each of these techniques will be discussed fully in subsequent sections of this whitepaper.

### Process Variables

Since 1993, substantial progress has been made by the pharmaceutical industry in the development of robust manufacturing processes. Techniques such as PAT are becoming more common in process control of manufacturing operations and in continual feedback and feed-forward loops that adjust manufacturing operations, thereby providing a more consistent end product. In parallel, improvements to end product testing results, including enhanced statistical tools, have further increased the ability of companies to manufacture more consistent products and to monitor and control variation. Today’s pharmaceutical scientist also has greatly improved statistical design tools with which to proactively develop and test formulation and manufacturing process parameters. Our collaborators have elaborated on these control strategies in several sections of this whitepaper.

### *In Vitro* Tests

*In vitro* testing was common in 1993 and was regarded as “a basic quality control tool used along with stability data to control scale-up and post-approval changes” (1). In this whitepaper, we review modernization of those techniques and the testing equipment used to monitor *in vitro* drug release, with an end goal of facilitating the development of IVIVCs that can be used to expedite post-approval changes.

### *In Vitro*/*In Vivo* Correlation

The 1993 Workshop Report on extended-release dosage forms stated that “in order to utilize an IVIVC, the adequacy of the *in vitro* method to act as a surrogate for *in vivo* testing must be demonstrated” (2). In the two decades that have followed, a number of IVIVCs have been developed. The current state-of-the- art approaches to IVIVC development are reviewed within this whitepaper in order to encourage further development of this important tool.

In addition to providing a brief review of all of the major tools in place today that are useful to the pharmaceutical scientist involved in the development of immediate- and extended-release oral solid dosage forms, our whitepaper contributors have included brief commentary on what they see as developing trends that could bring new exciting tools to bear on these issues in the next 3–5 years.

## CURRENT PRINCIPLES THAT AFFECT IR AND MR PRODUCT DEVELOPMENT

### Introduction and Current State

The Federal Food, Drug, and Cosmetic Act and the Code of Federal Regulations (Section 314.70(a)(2)) state that the applicant holds the final responsibility for determining the effects of a change on the drug product as it relates to the product’s safety and efficacy before distributing the drug product made with a manufacturing change. When the safety aspect of the product is brought into question as a result of a change, a prior approval supplement is called for, irrespective of the suggested filing category for that change. Other than for minor editorial changes such as spelling corrections or reformatting batch records, etc., the applicant must notify the FDA about each change that is made beyond the range that is allowed for that change in the approved application (Section 314.70(a)(1)).

A Supplement or Annual Report must include a list of all changes contained in the document. The list must describe each change in sufficient detail so that the agency can make an objective assessment on the appropriateness of the reporting category used. For supplements, the list must be provided in the cover letter (Section 314.70(a)(6)); for annual reports, the list should be included in the Summary Section (Section 314.81(b)(2)(i)). Each change must be described in complete detail inside the document.

Available post-approval regulatory documents in the USA cover a range of topics, but are more focused on drug product and changes associated with drug product manufacture. There are some regulatory guidance documents in the USA that cover drug substance changes, but are fairly limited in scope. A few FDA guidance documents issued were specific to drug substance, e.g., Bulk Active Compound Post-approval Change (BACPAC) 1, but have since been withdrawn. Thus, there is a pressing need for active pharmaceutical ingredient (API)-focused guidance documents for post-approval changes in the USA.

Changes to the drug substance control strategy may be subject to post-approval change requirements, as stipulated in FDA guidance documents. For example, changes to the drug substance manufacturing process require the submission of a prior approval supplement as defined under computer-assisted NDA (CANDA) requirements, unless the change is covered by an approved design space in the New Drug Application (NDA).

The availability of ICH Q7 and Q11 guidance documents has relieved the current gap in a limited way. However, post-approval changes and submissions pathways to handle those changes are quite regional in nature, and these guidance documents do not address that issue. Several guidance documents have been issued by non-US regulatory agencies [e.g., EC communication on variations (3) and Health Canada guidance on post-notice of compliance (NOC) changes (4)] that offer valuable insight into the regulatory pathways (ex-US) to submitting post-approval changes.

### ICH Initiatives (Q8, Q9, Q10, Q11)

The FDA’s Critical Path Initiative (5) triggered the development of new quality paradigms in the pharmaceutical industry, including the concept of QbD. The FDA’s ultimate goal was to transform its CMC review practices into a science and risk-based pharmaceutical quality assessment system which could potentially lead to an increase in the number of successful new applications and a reduction in the number of post-approval supplements.

Specifically, the FDA’s objectives were to:

- Encourage early adoption of new technological advances by the pharmaceutical industry
- Facilitate industry application of modern quality management techniques, including implementation of quality systems approaches, to all aspects of pharmaceutical production and quality assurance
- Encourage implementation of risk-based approaches that focus both industry and the agency attention on critical areas
- Ensure regulatory review and inspection policies are based on state-of-the-art pharmaceutical science
- Enhance consistency and coordination of the FDA’s drug quality regulatory programs, in part, by integrating enhanced quality systems approaches into the agency’s business processes and regulatory policies concerning review and inspection activities

These objectives are reflected in the ICH’s Q8, Q9, and Q10 guidance documents.

Any discussion on QbD necessitates defining pharmaceutical quality, which is rather subjective. ICH defines drug quality as the ability of a product to satisfy stated needs, including identity, strength, and purity, without undesired side effects (6).

Historically, the relationship of product quality to product attributes has not been well defined or understood. As a consequence, the FDA has ensured product quality via tight specifications based on the observed properties of clinical and/or technical batches and by limiting the changes that can be made within manufacturing processes (7).

In considering post-approval changes to an approved NDA or abbreviated New Drug Application (ANDA), current guidelines do not readily allow for the consideration of risk-based and science-based approaches for regulatory decision making. The guidelines were not developed based on a thorough understanding of the manufacturing process, prior knowledge and experience from similar types of products, and overall quality in determining whether a submission is required. Current guidelines recommend manufacturers to assess the effects of manufacturing changes on identity, strength, quality, purity, and potency of a drug as they relate to the safety or effectiveness of a product.

This rather prescriptive approach has contributed to pharmaceutical companies being reluctant to change their manufacturing processes and equipment from time to time from a continuous improvement perspective. The current SUPAC Guidance for Immediate- and Modified-Release solid oral dosage forms permits manufacturers to determine the submission requirements and category based on a predefined algorithm of information and data.

The introduction of the ICH Quality Guidelines, Q8 (R2), Q9, and Q10, and Q11 are intended to help develop a science- and risk-based approach to quality and, at the same time, encourage continuous improvement as part of a product’s life cycle through an effective pharmaceutical quality control system.

Furthermore, the FDA’s Manual of Policies and Procedures (MAPP), specifically MAPP5016.1, outlines and clarifies how the CMC reviewers in the Office of New Drug Quality Assessment in the Office of Pharmaceutical Science should apply the guidance recommendations to the review of regulatory submissions (8).

Since the ICH Guidance documents have been reviewed extensively elsewhere, they are assumed to be part of the working knowledge of our readers and will not be reiterated within this whitepaper other than to list their main topic areas, namely,

- ICH Q8 (R2)—Pharmaceutical Development (9)
- ICH Q9—Quality Risk Management (10)
- ICH Q10—Pharmaceutical Quality System (11)
- ICH Q11—Development and Manufacture of Drug Substances (Chemical Entities and Biological/Biological Entities) (12)

The concept of design space for pharmaceutical manufacture was suggested in the Guidance for Industry—PAT—A Framework for Innovative Pharmaceutical Development, Manufacturing and Quality Assurance (13). The definition of design space was provided in ICH Q8 (R2) as a multidimensional combination and interaction of input variables such as material attributes and process parameters that have been demonstrated to provide assurances of quality. Working within such a design space should not be considered a change. The identification of the material attributes and critical process parameters is important in describing the boundary of a design space.

Movement outside a design space is generally considered a change which would normally initiate a regulatory post-approval change process. Using the principles in ICH Q9, the level of risk when assessing a change scenario requires sufficient information and detail on how the risks were identified, characterized, and evaluated to clearly convey a full understanding of the decision-making process and the impact of any subsequent decision in a given product or process change. Combining these risk principles to assess control, communicate, and review quality risks, with prior knowledge and understanding, it should be possible under the principles of a robust quality system as outlined in Q10 to determine, using sound scientific knowledge and judgment, the potential impact on patient safety and efficacy.

This should provide opportunities for determining the appropriate regulatory approach to a given product or process change and still meet the requirements as outlined in the 21 CFR 314.70 which outlines current requirements for changes to an approved NDA or ANDA (Prior Approval Supplement, CBE/CBE-30 or Annual Report) (14).

### FDA Guidance

#### Changes to an Approved NDA or ANDA

An FDA-issued guidance (15,16) makes recommendations for post-approval changes in components and composition, manufacturing sites, manufacturing process, specifications, container closure system and labeling, as well as multiple related changes. This guidance does not provide recommendations on specific information required to assess the effect of changes to identity, strength, purity, or potency of a drug product. In general, it discusses three types of changes: major changes which have a substantial potential to have an adverse effect on product quality attributes of identity, strength, quality, purity, or potency as they relate to the safety and efficacy of a drug product; moderate changes which have a moderate potential to affect product quality attributes; and minor changes which would have a minimal potential to adversely impact product quality attributes.

#### CMC Post-approval Manufacturing Changes Reportable in Annual Reports

In an attempt to reduce the number of manufacturing supplements in recent years, in connection with FDA’s Pharmaceutical Product Quality Initiative, and incorporating a risk-based approach to the CMC review process, a list of post-approval manufacturing changes (16) that can be reported in an Annual Report was developed. This listing includes changes to components and compositions, manufacturing sites and processes, along with specifications, container closure systems, and miscellaneous changes.

### Canada, Europe, Japan, and the Rest of the World’s Countries

While the SUPAC Guidance is specifically considered a US-only guidance, it is viewed favorably by some other regulatory authorities, and aspects of it are incorporated as part of their country-specific post-approval change regulatory requirements.

As many companies develop products with a global perspective, there are other guidance documents that may be considered when making post-authorization changes to approved immediate- and modified-release products.

#### Canada

##### NOC Changes—Quality Guidance Appendix 1 for Human Pharmaceuticals, Canada Ministry of Heath, Health Products and Food Branch

This guidance, implemented 17 October 2011 (4), outlines the categorization of post-approval changes and makes recommendations for supporting documentation based on the level of risk. This revised document simplifies the process for submitting post-authorization changes in manufacturing and chemistry which require Health Canada approval. This revision eliminates level II (Notifiable Change) and moves higher-risk submissions to level I while moving lower-risk changes to level III. The intent of this revision was to provide greater clarity for filing requirements of supplements without affecting safety and efficacy.

The data requirements associated with the changes outlined in the PNOC-QD are based on (commensurate to) the level of existing knowledge and established assurance of quality of a product afforded by an NOC and, in many cases, also by market manufacturing experience.

Where there are differences between Health Canada data requirements to support pre- and post-NOC changes, sponsors are expected to include information and data in their submission in accordance with pre-NOC guidance documents (17,18) rather than with the PNOC-QD.

#### European Medicines Agency

In the EU, amendments to an approved application are called variations. In 2003 (19), the Variations Guidance was amended; the intent was to simplify reporting procedures and, at the same time, provide the same regulatory framework for changes in both the mutual recognition and the centralized procedures.

There are three categories of post-approval variations (3). Type 1A variations are considered minor changes. Such changes are simply administrative in nature or other modifications which do not affect the quality, safety, or efficacy of the product. There are two subcategories of 1A variations, which have clearly defined document requirements and set of conditions for the change

Type 1A variations are “do and tell.” Generally, the IA variations can be submitted within 12 months of implementation. These 1A variations will be equivalent to the US annual reports. There is also a type IA(IN) variation where immediate notification is required upon implementation. This is similar to the US CBE.

Type 1B variations require notification of changes before implementation. Regulatory approval is expected within 30 days of submission for changes that are considered more scientifically minor in impact. Type 1B variations are given as examples rather than as set criteria and conditions. There is a 1B default classification for changes that are neither a 1A nor a type II variation. The idea behind type 1B changes is to create flexibility for industry, but with that flexibility come the challenges due to the lack of clearly defined requirements. Type 1B variations are essentially equivalent to the US CBE 30.

There is also an “unforeseen” variation approach for those variations that are not listed in either the guideline or the regulation and for which the type IB by default is uncertain or controversial and believed to be a lower category than a type II variation. The Ministry of Health may request the CMDh (Co-ordination Group for Mutual Recognition and Decentralised Procedures—Human) to provide a recommendation on the classification of the variation according to Article 5 of the regulation. Timings of this process can be equivalent to timings of a type II change, so one really needs to make sure there is a need to have a change classified.

Type II variations are for major changes not meeting the criteria for minor variations and line extensions. Type II variations are the severest category and will be prior approval change, similar to the US SNDA. The approval timing for a type II variation is typically 3–6 months.

In an attempt to further simply these variation classifications, the European Commission (EC) has proposed current amendments to the current regulations (20).

One difficulty in dealing with the EU is that there are a number of different marketing approvals that exist in Europe, i.e., centralized, decentralized, and national, and the administrative reporting burden varies with the different approval processes.

#### Japan

Japan revised its pharmaceutical Affairs Law in 2002 which amended its procedures for post-approval changes. Depending on the nature of the change, the requirements can be quite extensive, with a lengthy review time. Items noted as minor do not require pre-approval and can be submitted as notifications (21).

### Quality By Design Principles

#### Importance of TPPs and QTPPs in Product Development

##### Proactive Identification and Definition of Desired Target Product Quality

A quality target product profile (QTPP) relates to the required quality of a drug product or drug substance that is necessary to deliver a desired therapeutic effect. When prepared proactively, the QTPP serves as a set of predefined objectives establishing product and process deliverables that will provide the greatest benefit for attaining the critical attributes that impact drug product quality. These predefined QTPPs evolve over time during drug development and may be modified to incorporate new knowledge, as is warranted by ongoing clinical studies; such as dose effect and toxicology data that are often conducted in parallel with a product’s developmental activities.

##### Clinical, Patient, Marketing Alignment

The QTPP comprised all the necessary attributes a product needs to deliver in order to meet its intended use. A product’s QTPP provides guidance for linking its process, formulation, and incoming materials with therapeutic patient outcomes. Examples of clinically relevant QTPPs include, but are not limited to, dosage form and biorelevant data, dosing regimen that achieves a predefined clinical result, pharmacokinetics (PK), bioequivalence, and established efficacy and safety profiles (9). In order to maximize ultimate product performance, other attributes of a drug product may also be considered, such as product identify (tablet size, shape, and color) and intended patient population.

##### QTPP and Risk Assessment

Once all of the components of a QTPP have been defined, a preliminary risk assessment should be completed before starting development activities. This allows for revision of the QTPP, if needed, and guides development activities prior to investment of major development resources. It is important to note that risk assessments should be updated as further knowledge is gained during development.

#### Importance of CQAs in Product Development

##### Align with QTPP

Once a product’s QTPP is defined, the appropriate formulation and process characteristics of the product can be developed around it. The attributes that assess the quality of the drug product or drug substance aligned with the QTPP are referred to as the critical quality attributes (CQAs). They link the product’s quality with its desired therapeutic performance. Similar to QTPPs, when CQAs are proactively identified, establishment of target process deliverables can be prepared and alignment with therapeutic targets achieved. Where QTPPs are derived based on desired clinical responses, CQAs are those characteristics that reflect the final process and product quality. For example, where bioavailability is an element of a product’s QTPP, dissolution may be its associated CQA. Although the CQAs are deemed product quality- and/or process-relevant, it is important to understand how they can potentially affect patient therapeutic outcomes when establishing ”acceptable” specification ranges. In this manner, the QTPP guides the selection of appropriate CQA specifications (or specification ranges) based on those critical attributes ultimately necessary for delivering the desired patient effect.

Changes made to the QTPP during development will necessitate corresponding changes to the product’s critical manufacturing attribute(s) (CMAs), critical process parameters (CPPs) and CQAs.

##### Process-Delivered Responses

The CQAs that define the drug product QTPP typically include assay, dissolution results, stability, impurity profile, microbial burden, and physical appearance. Presently, CQAs are similar to the drug product and drug substance release and stability specifications as they traditionally reflect the desired patient deliverables. However, CQAs are potentially influenced by multivariate parameters, which themselves may interact with other parameters or material attributes throughout the manufacturing process. Therefore, as the development process evolves, new knowledge may be uncovered that challenges earlier conclusions. This requires that CQAs are updated throughout the product development processes. An example is the possible shift of a CQA from dissolution to disintegration time (DT) for an immediate-release (IR) dosage form. As development proceeds and additional knowledge gained, it may become apparent that DT is a better quality surrogate than the traditional dissolution.

##### Correlation to CPPs

Process understanding through identification of potentially influential variables (such as process parameters, material attributes, drug substance quality, etc.) on CQAs is an integral aspect of the pharmaceutical development process. Those variables that have an impact (i.e., elicit a significant response) on a CQA(s) are deemed “critical.” When assessing criticality, it is helpful to have predetermined criteria for significance. For example, is a 3% impact on dissolution of an IR or modified-release (MR) product considered significant or can the impact be explained by the inherent variability of the analytical method? In-process attributes may be more challenging to define. Examples such as blend or granulation flowability (via Carr index or Hausner ratio), while easy to quantify, may be difficult to establish as a predetermined critical metric, especially if such attributes are not commonly monitored/studied and therefore no basis of comparison is available. It is important for the formulation scientist to evaluate how much variability in the associated attribute could elicit a response which can ultimately impact product quality. Understanding the main effects that critical process parameters, material attributes, and their mutual interactions have on a product’s defined CQA(s) is important in order to establish control of the CQAs and, ultimately, the final product quality (22).

#### Importance and Demonstration/Determination of CPPs and Resulting Design Space

##### Correlation and Alignment to Specific CQAs (Cause/Effect Relationship)

CQAs provide the link between critical formulation and process parameters with clinical product performance. Therefore, all critical sources of variability inherent in a formulation or process should be identified and understood and a rationale established on how they will be managed going forward.

Once an understanding of critical process parameters and material attributes is established for those variables that affect respective CQAs, deeper knowledge can be developed regarding the magnitude of effects. Ultimately, this helps establish the appropriate ranges of the multivariate design space. In order to attain this level of cause–effect understanding, one needs to establish a correlation of the CPP and/or CMA with its associated response on the design space. This becomes even more relevant when striving to attain real-time release testing (RTRT), where surrogates for therapeutic performance are correlated not only with analytical release tests but optimally to a specific process parameter(s) or material attribute(s) that impacts that analytical response. It is in these cases where mitigation of risk and optimal delivery of product performance can be delivered because the direct influence on quality can be measured. Additional information on RTRT can be found in subsequent sections of this paper.

##### Identifying Current Process Risk and Variability Using Systematic, Multivariate Design of Experiments and Cross-Discipline Team

The risks associated with a product, whether during development or commercial scale, are tied to the amount of incoming variability caused by materials and process conditions (i.e., parameters, equipment scale, means of operation, etc.). Distinguishing critical parameters from those variables whose impact is minimal or totally insubstantial is the essence of pharmaceutical development. Cross-functional teams composed of a wide array of disciplines (i.e., process development experts, formulators, statisticians, analysts, technicians, etc.) provide a varied perspective on the important formulation risks.

Traditionally, criticality evaluation has been performed by varying one condition at a time. Using this methodology, experiments are conducted by varying one parameter while keeping all others constant (fixed) and then assessing the response (impact). This manner at which variable performance is studied one factor at a time (OFAT) is not necessarily how parameters behave during routine production. The shortcoming of this methodology is that it fails to provide the experimenter with an understanding of interactions between variables. Oftentimes, these interactions go unnoticed and can result in synergistic affects when multiple variables are allowed to vary collectively, thereby resulting in a magnitude of effect that renders the initial parameter range suboptimal.

In contrast, the design of experiment (DoE) approach is designed to evaluate systematic variation of multiple factors (i.e., variables) within the context of one experimental design. DoEs can, thereby, be used to identify criticality of variables and ultimately create mathematical models of the process being examined aimed at predicting process performance (23).

Once a relationship between a CPP and CQA is demonstrated, the variable that demonstrated impact on a CQA should be further evaluated with the goal of understanding how much variability can be induced (i.e., how far the parameter range can be) in relation to other parameters as they collectively deliver acceptable product quality. Finding the technical edge of failure is not always necessary when evaluating criticality factors, especially when the range of the factor is unrealistically wide as it will likely not be employed in production. Therefore, when constructing a DoE, it is important to keep in mind the level of desired variability induced in order to learn as much as possible about a process within the given resource constraints.

**Fig. 1:**
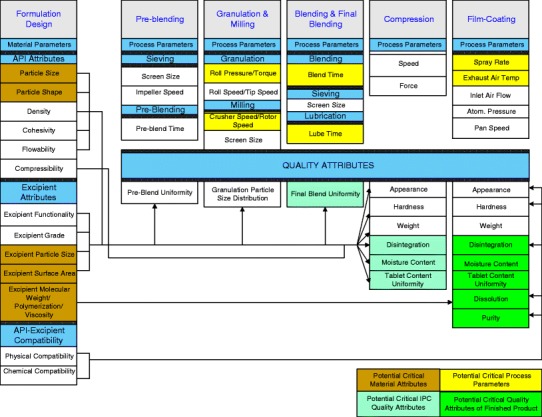
Relationship between formulation design factors and CQAs

The complex relationship between formulation design factors, manufacturing unit operations, and their corresponding quality attributes is shown in Fig. 1. It is the responsibility of the development team to identify, and set control limits for, the attributes that are found to be critical to the quality of the product (22,23).

##### Assessment of Significance

Statistical significance results when the factors of a study cause an impact to the identified CQAs. It should be noted that although variables can have statistical significance, the practical significance must also be evaluated. If a sample size is large enough, variables will undoubtedly demonstrate statistical significance. The scientist must evaluate what practical meaning this effect has on CQAs.

One way of accessing practical significance is to evaluate the magnitude of effect the critical variable has on a given CQA. For example, if particle size distribution is statistically significant, it should only be deemed critical if the magnitude of the affect is large enough to be of practical concern. This is where establishing predetermined criteria for significance becomes valuable (as previously discussed).

Prior to the DoE and execution, the scientist should clearly define how much variability in particle size distribution is considered practically significant, in that the variability over this predetermined range would likely have an impact on drug product quality. While determination of statistical significance may be generated from statistical packages (i.e., SAS, JMP, MiniTab, etc.), practical significance determination should be made with the CQAs and patient effects in mind. While a variable may significantly impact an in-process attribute (i.e., particle size distribution, density, flow, etc.), the final CQAs may not necessarily be impacted. This is commonly seen with particle size distribution of granulation that may be impacted by variability in upstream conditions (i.e., granulator speed, time, etc.), but ultimately the overall manufacturing process (such as milling, blending operations) is robust enough to mitigate or eliminate these effects and no ultimate effect is demonstrated on final product quality.

##### Combining Input Variables and Process Parameters that Have Been Demonstrated to Provide Assurance of Quality

Prior knowledge, proactive team-based risk assessments, multivariate experiments, use of PATs (discussed in later subsection), and continual improvements are used to develop process understanding to a level necessary to establish a design space for a drug product or drug substance. The design space reflects the product understanding as a multivariate area where the impact on product quality is understood and controlled based on the variability of critical processing parameters and/or material attributes within a known range. A distinction between a design space and proven acceptable ranges (PARs) should be clearly made. PARs lack the capability to provide understanding of potential interactions among other parameters throughout the drug product manufacturing process. These interactions (either synergistic or antagonistic) can potentially affect a process and may not necessarily be determined if studied one at a time in a univariate manner.

Essentially, the manufacturing process runs in a multivariate environment (i.e., numerous factors are simultaneously in operation). Therefore, the parameters of that process should be studied in order to fully understand their effects on the design space.

#### Defining an Appropriate Control Strategy

##### Mitigating Risk Through Maintenance of CPPs and Adherence to Design Space to Ensure Product Quality

Once a process is developed and transferred to commercial scale, generally, the scope changes from inducing process variability in order to elicit an effect to monitoring the normal variability in the process and establishing/maintaining process control. Critical parameters that make up a design space should be controlled and continually monitored for trends and shifts that could occur over time in order to avoid significant deviation which could ultimately impact product CQAs. The design space is held within specifications via a control strategy, which is defined as “a planned set of controls, derived from current product and process understanding that ensures process performance and product quality” (9).

The establishment of a control strategy is necessary to ensure that the compliance of the drug product or drug substance processing parameters is delivered and that material attributes are consistently met and remain within their predefined settings. It is important to note that the control strategy should be developed specifically for the individual process that it monitors. A control strategy should include appropriate elements that will provide monitoring and/or controlling potential positioned at strategic junctures of a process where criticality has been demonstrated. Typically, a control strategy includes items such as additional in-process testing, use of PAT tools/probes, IT/software programs (i.e., feedback loops and automated recipes), well-defined batch records, specifications, etc. For example, if incoming raw material variability has been demonstrated to be critical to a CQA [i.e., disintegrant, particle size distribution (PSD) impact on dissolution], a supplier change could alter the incoming PSD characteristics that affect CQAs even though routine supplier sourcing evaluation is followed and specifications met. It is in this manner that true process monitoring is attained and, if performed far enough upstream, intervention possible.

##### Means of Constant Process Observation Using Technology and Science

**Multivariate Data Analysis**: As previously mentioned, criticality is determined by assessing the magnitude of impact a variable (parameter or material attribute) has on a response (CQA). Therefore, the relationship between the parameter and CQA needs to be understood. Multivariate data analysis (MVDA) is a form of statistics that helps to understand the relationships between variables, observations, and their relevance to each other (using principal component analysis, PCA) as well as relationships between variables and responses (using partial least squares, PLS). When coupled with process knowledge and criticality understanding, PLS and/or PCA models can be used to construct multivariate statistical process control (MSPC) charts in order to identify deviations from targeted behavior. MVDA uses established algorithms to create linear models comprising an approximation function and level of concomitant noise. MVDA models are designed to assess and ensure (in near real-time) that the progression of a batch is evolving within the defined design space during processing, thereby ultimately yielding material meeting predefined critical quality attributes. By this methodology, the process parameters are summarized by a few critical variables (“scores”) instead of a vast number of individual process parameters with limited significance.

Generally, these variables are defined by production of a series of target batches manufactured within normal operating range (NOR) parameters. NOR conditions are generally tighter than the encompassing design space and represent the day-to-day parameter settings where slight variability is allowed. Since the behavior of these batches is understood and even desired, target batches are normally used to build the reference model. Batches that were produced in other areas of the design space, generally at greater extremes, are either excluded from batch monitoring models or used to challenge the model for sensitivity. Consequently, batch quality can be depicted graphically and deviations of process variables from their expected range can be visualized online in real time, allowing for immediate attention and adjustment.

##### Process Analytical Technology

Process analytical technology (PAT) is defined as “a system for designing and controlling manufacturing through timely measurements (i.e. during processing) of critical quality and performance attributes for raw and in-process materials and also processes with the goal of ensuring final product quality”(13). PAT tools include spectrographic equipment such as near-infrared (NIR) and Raman, but can also include software enhancements that lead to a greater understanding of process execution. Some examples include power consumption, *ΔT* (difference in drying temperature used in wet granulation), and the MSPC models described above. The one element these examples of PAT have in common is their connection to the dynamic process data of a specific parameter and resulting data. These technologies allow for innovative development means by providing fundamental understanding of drug product processes.

Advances in technology have made available many tools that provide effective means for acquiring information to facilitate scientific understanding, continuous improvement and development of risk-managed pharmaceutical development, manufacture, and quality assurance. One of the most common PATs is the utilization of multivariate tools for design, data acquisition, and analysis: For example, design of experiments and multivariate data analysis seek to measure the interactions between multiple process variables simultaneously. Interactions between process variables are the most frequent attributable cause of process failures and are not typically detected, and thus a major pitfall of the univariate design. When used appropriately, multivariate tools enable the identification and evaluation of product and process variables that may be critical to product quality and performance. These tools may also identify potential failure modes and mechanisms and help quantify their effects on product quality. Therefore, PAT is part of an overall strategy of continuous improvement that enables continuous learning through data collection and analysis over the product life cycle.

##### PAT Process Controls, Controls Integration, and Information Management

**Process Controls and Model-Based Systems**: In today’s highly automated manufacturing environment, parametric controls are a critical component of the process control strategy and are fundamental to the process information and knowledge base.

Multiple components in pharmaceutical manufacturing machinery have elements of performance control, monitoring, and measurement; however, only the relevant control elements that are influential to CQAs are typically included as part of the process control strategy.

The evaluation of the equipment–product interaction is a key element of risk management analysis. Analyzing equipment–product interaction as part of the risk management tools allows for the determination of what is important to measure and control. ICH guideline Q9 provides risk analysis tools that can be used in the determination of the equipment–product interaction risk elements.

**Fig. 2:**
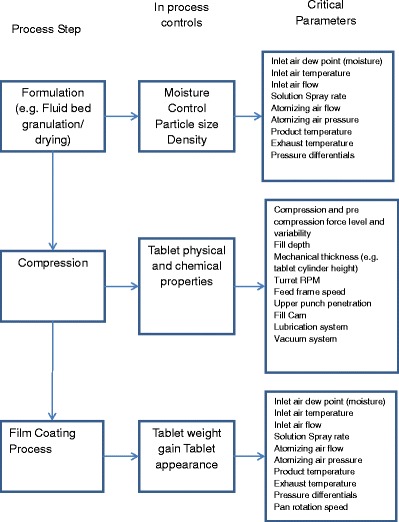
PAT control elements of a typical solid dosage form manufacturing process

Typically, a pharmaceutical manufacturing process has equipment components that fit the need for the type of control design, either discrete, batch, continuous, or a combination of them. Figure 2 shows a diagram of the equipment train of a typical solids manufacturing process. Note that each piece of equipment in the figure includes a list of the equipment parametric control elements that are commonly agreed as process performance or safety controls. Filtering the quantum of parametric information into a critical parameter list requires the analysis of the equipment–process interaction with respect to available process knowledge and the criticality analysis on product quality. The criticality analysis of the process parametric space results in a failure mode hypothesis that needs empirical confirmation. The empirical confirmation of the failure modes often requires experimental evaluation, particularly in a new product or process design; otherwise, historical data or a combination of DOE and historical data shall lead to the definition of the critical process parameters.

Upon DOE execution, the hypothetical failure modes and their control definitions are either confirmed as critical or regarded as noncritical for the product CQAs. The conformance of the quality attributes satisfying the target product profile expectation may then be defined in a design space relevant to critical (equipment) process parameters (CPPs) that become part of the equipment control strategy.

The availability of a multivariate DOE-based process fingerprint shall be used in the establishment of process empirical models that relate relevant CPPs to the product CQAs. The initial goal for the use of the DOE data is to establish control loops, or limits of manual adjustments by equipment operators. In particular, when combined with CQA-surrogated process analytical technology (e.g., NIR, Raman, or fluorescent spectroscopy), the empirical model can become a process/product performance predictive model with real-time release capabilities that could enable dynamic control strategies. An expected subsequent improvement of the control strategy should include the analysis of process relationships to fundamental theoretical models that explain the observed process performance with mathematical accuracy (e.g., first principle and thermodynamic models, population balance models, mass balance models, diffusion models, etc.). The integration of the empirical data and the fundamental mathematical modeling along with the in-process analytical technology is a powerful tool that should become the ultimate goal of the control strategy when possible. This is particularly true when material quality attributes are well established and controlled and previous knowledge regarding model systems relevant to the pharmaceutical manufacturing process are considered.

**Controls Integration and Information Management**: The process control inputs, when defined relevant to the CQAs, are part of the PAT toolset and contain statistically significant predictive information that should be monitored and used as part of the control strategy. Univariate parametric information can be established as a first line of defense against process variation that could be detrimental to product performance. Charting techniques using statistically defined limits (e.g., run charts, moving average, moving range, or Shewhart control charts) can serve this purpose. Multivariate analysis of parametric data can also be important. Multiple regression analysis, PCA, and partial least squares or projections to latent structures (PLS) are tools available for multivariable data analysis. Charts of these data, either by score loading charts (PCA or PLS), residuals analysis, control or run charts of the product of the multivariable function, can provide adequate control monitoring for the multivariable relationship, allowing process parameter adjustment that is capable of restoring the process balance in case of drift conditions. Examples of processes that are multivariable in nature include wet granulation process, fluid bed granulation, fluid bed drying, roller compaction, compression process, film coating process, etc.

The equipment controls system SCADA (Supervisory Control and Data Acquisition) allows for data evaluation, calculation, and display using graphical tools that should be presented to the operator using a human–machine interface (HMI). This information provides a means of alerting the operator to deviations from the expected parametric performance. Upon an observed deviation, there should be in place deviation definitions and control policies (i.e., actions that should be taken to reestablish the desired process outcome) based on the defined CPPs and within the design space established via DOEs. The control strategy can be based on open loops, which require the operator to be trained and have the necessary skills to perform a process correction. On the other hand, a closed-loop control schema can be established to automate the response to the drift condition and to restore the process outcome independent of any operator action. The control action can be a feedback action upon the control, affecting the output of an upstream process step or equipment, or a feed-forward action, for which the measure of the output of the preceding step is used to define processing conditions of downstream process steps. Similarly, the analysis of intermediate material or final product property measurements using CQA-surrogated PAT (e.g., NIR, Raman, LIF, or other tests) should be considered in the control loop strategy, either as open or close loops. Under the scenario where the feedback or feed-forward control loop is based on an analytical surrogate in-process test, it is particularly important to ensure the adequacy of the measurement system, including sample presentation (e.g., sample size, probe location, accuracy, and reproducibility of measure), control loop response time, and define the targeted control strategy (trajectory tracking, midpoint correction, or drift correction).

##### Life Cycle Management Through Proactive Process Enhancement/Optimization

The established design space is required to be monitored throughout the life cycle of the product. For example, annual product reviews (APRs) are conducted on a yearly basis to assess the consistency of the quality production. Based on the new information generated during ongoing quality monitoring activities, any necessary improvements and changes needed to address trending and/or process optimization potential should be implemented as described in local quality systems concerning change management.

Annual product reviews are industry standards for assessing batch data over the yearly production activities. CQA data are evaluated for shifts as well as variability both over normal production as well as any post-process optimization implementation initiatives that may have taken place. Statistical assessment of data can provide a more representative account of CQA variability. Statistical measures of process quality capability, such as ANOVA, Cpk, and Ppk, assess variability around a mean and can therefore quantify the magnitude of variability of release data. DoE batches would not be used to calculate process capability because of the induced variability a DoE introduces to a process for the purpose of impact assessment. Cpk is designed to evaluate normal variability of a process (i.e., at set point or target conditions). Although CQA impact assessment provided by a DoE is beneficial from a CPP establishment perspective, the batch results should not be used for Cpk calculation because the inherent process variability cannot be assessed accurately. When none of the DoE parameters studied have an impact on a CQA, then all the DOE batches can be assessed collectively, as noncritical parameters. Hence, only the batches that were made with the same process conditions (i.e., target batches) are included in the Cpk calculation.

Another component of life cycle management is the utilization of continued process cerification (CPV) to proactively monitor routine production. The goal of continued process verification is to assure that the manufacturing process continuously remains within a predetermined state of control during routine commercial manufacture. Through CPV, unplanned deviations as well as trends in input variables, in-process control (IPC) results, and final product quality are detected and an assessment made regarding necessary improvements throughout the life cycle of the product.

A successful CPV program has systems in place which have the ability to proactively identify potential issues before they become critical. For example, statistically evaluating analytical release data which may be within specification but trending low or increasing in total variability can result in the avoidance of an ultimate failure if the cause of variability can be discovered and rectified prior to it reaching a significant magnitude.

Statistical process control techniques and data collection plans may also assist with the evaluation process as to the cause of variability. Statistical analysis of release and in-process data coupled with in-depth process knowledge obtained through risk assessment and criticality analysis can provide the means of identifying opportunities to optimize the current process and/or identify areas where enhanced detection mechanisms are needed to ultimately improve end product quality.

##### Use of PAT in Supporting Post-approval Changes to IR and MR Products

Quality risk management is a key part of PAT implementations by identifying potential quality concerns and implementing appropriate controls. The combination of risk management and PAT has allowed the ability not only to identify potential quality concerns but also implement ways to improve the likelihood of detection and/or control of the product/process. This section discusses PAT applications, how changes can be made to PAT applications, how they can be added or removed from processes, and how they can be used to support post-approval changes to an IR/MR product. Rather than identifying specific controls required, examples are provided and quality risk management is used to guide the appropriate requirements for changes.

**R&D Requirements for a PAT Application In Order to Support Post-approval Changes**: As part of product and process development, PAT methods can be used to support process understanding and facilitate scale-up and commercial site transfer. This information would be included in the filing as part of the process justification. The methods used for process understanding or design space development may not be validated or required for commercial manufacturing. Existence of these methods and reference to them in the filing may facilitate using the methods again for a site transfer, additional scale-up, or other post-approval changes. It is expected that PAT methods utilized in product/process development may support the post-approval change through inclusion of previous data and greater process understanding.

There are also scenarios where an online or at-line method is developed during development as a surrogate for a traditional high-performance liquid chromatography (HPLC) test. However, due to limited number of batches manufactured during development, the method would require additional updates before full commercial implementation. As defined by the PAT method maintenance strategy, after the product is approved and commercial manufacturing begins, data from commercial batches are used to update the PAT method and demonstrate its capability to support the commercial-scale process as intended. Assuming the defined outcomes are achieved with regard to predefined acceptance criteria, online/in-line might then become the primary commercial test method.

It is possible to have an in-process PAT method introduced to measure an attribute at an intermediate step and eliminate the need for the measurement of that attribute in the final product. In this case, it is not a direct substitution but is a way of testing for a product attribute at the appropriate manufacturing step and enables a feed-forward or feedback control loop that could assist with achieving consistent product quality by adjusting the appropriate process parameter(s). PAT measurements at intermediate steps reduce product risk because they allow for a process control and parameter adjustment to ensure the final product meets a predefined quality specification. Such a control scheme would also require sufficient commercial-scale data prior to full implementation. This may require, similar to what is mentioned above, completion of method development by the development team and method inclusion in the filed documentation.

For the two cases above, predefined acceptance criteria are needed prior to the filed submission and need to be agreed upon by all accountable organizations or departments. In addition, the methods proposed would be developed and validated through collaboration between the development and commercial organization. The expectation is that these methods would be consistent with the sponsor’s validation policy. In addition, discussion with the regulatory agency prior to the filed submission is recommended to insure alignment of expectations for the method validation, implementation, and maintenance.

**Support of Changes from Previously Filed PAT**: PAT methods are integral to continuous improvement and monitoring of a manufacturing process. It is expected that the PAT method will itself evolve and require continual improvement. As such, the quality system for the PAT method should include a procedure for updating the method with respect to increasing process understanding through greater process experience which captures variability associated with raw materials and non-product quality-related changes in the process. The requirements for updating the PAT method will vary depending upon the overall significance of the PAT method to the process control strategy and the assessed impact of the changes to method robustness. Simplified, the higher the criticality of the PAT method to a product CQA, the greater the expected level of detail for any changes to the PAT method. This criticality is best determined by reviewing established risk assessments. For example, a real-time release method, which is considered a direct substitute for a traditional laboratory release method, is a high-impact method, and changes to the method would be expected to capture the performance attributes critical to the CQA.

Complementary medium-impact methods (design space-related methods that are not sole indicators of product quality) and low-impact methods (for process understanding and development) would be expected to demonstrate performance attributes aligned to the intended use of the method.

Some level of expectation for PAT method evolution can be set at the development stage of the PAT method based on process and method development history. Ultimately, the PAT method maintenance strategy needs to be specified as part of the method’s quality system documentation at the site. A descriptive summary of the maintenance strategy should be included in filed documentation to align expectations for method evolution with agency guidance documents. In limited situations, where supported by a risk assessment, comparability protocols may be specified in filed documentation. Although, due to the complexity of comparability protocols, the preferred approach is to include the strategy for anticipated update(s) as part of the documented PAT method maintenance.

**Post-approval of New PAT Applications**: The continuous improvement and monitoring of a manufacturing process is likely to provide the opportunity to implement or improve PAT where it was previously not available or considered. The determination of the regulatory filing category should be based on the level of risk associated with the unit operation or attribute for which PAT is being utilized combined with the required filing category for associated changes. The following examples are provided to demonstrate the different degrees of risk and potential regulatory filing requirements.

1. The current blending unit operation for a solid oral dosage form includes blending of ingredients for a set number of revolutions at a set speed. In order to increase process understanding and gain additional knowledge about the blending process, NIR monitoring is added to the blending process to better understand blend uniformity throughout the unit operation. There is no change to the manufacturing process as the blending unit operation process parameters remain unchanged. The addition of NIR either maintains or reduces the current risk level and would not require regulatory approval.
  After gaining sufficient experience/data with NIR monitoring of the blending step, it is determined that controlling the completion of blending through NIR rather than a set number of revolutions ensures blend uniformity and a reduction in blending time. Therefore, it is desired to change the control of the blending step to utilize NIR. Additional continuous process verification would be added to ensure no change in the uniformity of dosage units CQA; therefore, the risk level is maintained or reduced. As a result of the change to the manufacturing process, the change would require regulatory approval (CBE/CBE30).
2. The process of spraying drug substance onto a core tablet is currently monitored using HPLC to determine the end-point prediction of the spray coating process. In addition, assay testing is performed at release for the finished product. A NIR method is developed to monitor drug substance growth on the core tablet, allowing rapid analysis and monitoring for the completion of spray coating. The level of risk is reduced due to the increased monitoring through the coating end point, allowing for a more precise end point determination. The change to the process control would require regulatory approval (CBE/CBE 30). After sufficient data are obtained using NIR to determine end point through a PQS-approved continuous process plan, it is desired to use NIR to replace finished product assay testing. The risk level would be maintained or reduced, and the change to specifications would require regulatory approval through a CBE/CBE30.
  In most situations, the implementation of PAT will result in either a reduction of risk or maintaining the current level, in which case prior approval (PAS) would not be required. This assessment is in line with the current Guidance for Industry Changes to an Approved NDA or ANDA (edition date, April 2004), which states “A change in methods or controls that provides increased assurance that the drug substance or drug product will have the characteristics of identity, strength, quality, purity, or potency that it purports or is represented to possess” be submitted as Changes Being Effected (CBE). The data requirements for the CBE should include submission of the PAT methodology and its validation. In addition, batch data demonstrating that the product manufactured with the new or improved controls continues to meet the existing specifications should be provided. The need for stability data should be dependent upon the degree of change to the process and the potential the change has to impact the stability profile of the product.

**Removal of PAT Based on Practical and Scientific Considerations**: PAT developed and implemented during product development stage serves as a key driver (or component) for rationalization of the manufacturing processes, knowledge-based production, and enhanced assurance of product quality after product approval (or launch). Following knowledge accumulation during commercial production, PAT may be removed or reduced under certain circumstances based on consideration of extent of product and process understanding, nature and role of the PAT, complexity of the product and process, availability, and life cycle cost of instrumentation. For instance, the following scenarios may prompt evaluation of possibly removing or replacing (or downsizing) PAT for commercial production:

- When statistical process control demonstrates that removal of the PAT does not result in a decrease in process capability.
- Change of manufacturing site where PAT transfer may be challenging due to instrument availability, need for equipment retrofit, method transfer, and cost.
- For products or processes that are controlled using a multimodal PAT, reduction of modality (e.g., to a single sensor) has been proven to provide sufficient (or equivalent) process control. In general, reduction, replacement, or removal of PAT is feasible (or can be considered) for a production operation that is fully under control, and
  - demonstrates robustness of the manufacturing processes and an understanding of the interplays between the process involved and raw material properties, scale, and type of equipment, etc.
  - shows consistent correlation between parameters of in-line or online analytical systems (e.g., NIR) and a non-PAT in-process control (e.g., number of revolution of a blending operation) within its design space.

When removing PAT, caution should be exercised for a complex manufacturing process and/or drug product, such as modified-release or amorphous solid dispersions, or where the replacement process control is purely empirical in nature (e.g., a combination of mixing time and liquid volume for determining the end point of a wet granulation process). Risk assessments should be revisited and updated to justify any reductions, replacements, or removal of PAT.

**Use of PAT in Supporting Post-approval Changes to IR and MR Products**: The principles for the SUPAC QbD approach are based on product-specific risk assessment of changes which may impact approved design spaces within the framework of the approved control strategy to ensure consistent product quality and performance. The changes and requirements are less prescriptive compared to the SUPAC IR/MR approach, although they require a detailed and documented understanding of the formulation and process with a focus on continuous learning and improvement. These general principles for application of the QbD approach include no requirements for regulatory notification for changes within approved design spaces. Under this latter scenario, all documentation to support the proposed changes would be managed through the PQS and be available for submission or review when requested. This documentation includes: changes to batch records, risk assessments with technical justifications to support process qualification testing, extended dissolution/bioequivalence requirements, stability and continuous verification plans. Use of the proposed QbD approach incorporating specific PAT in the control strategy is illustrated by several examples in Table I below for comparison to the traditional SUPAC IR/MR approach. The examples are general in nature, but specific scenarios under the general cases were developed in order to further demonstrate the QbD principles.

**Table I:** Use of PAT in Supporting Post-approval Changes

Change control element	Current SUPAC IR/MR	Proposed QbD approach
Change control management	Highly specified in guidance documents for most elements	Follow principles outlined in ICH Q10 for PQS.
Types of changes	Highly prescriptive, independent of product sensitivities	Product specific related to changes impacting design space and control strategy as outlined in ICH Q8(R2) and ICH Q11
Risk evaluation	Highly prescriptive based on level 1, 2, and 3 changes	Customized based on principles outlined in ICH Q9 for QRM. Example, assess level of risk such as FMEA tool based on prior knowledge and science with technical justification.
Filing documentation	Highly prescriptive based on level of change	No filing documentation required for changes within design space. Changes outside of design space would follow current regulatory requirements. May need to consider changes to any control strategy element from QRM approach
Manufacturing documentation	Specified for most changes	Managed through PQS
Application of compendial release documentation	Specified for most changes	Managed through PQS. May need to consider additional extended testing and validation requirements such as continuous process verification based on QRM approach
Stability documentation	Highly prescriptive based on level of change	Customized based on risk assessment and product sensitivities. May need to consider the impact of any change to a control strategy element that could impact stability-indicating CQAs
Dissolution documentation	Highly prescriptive based on level of change	Customized based on risk assessment and product performance sensitivities
		May need to consider the impact of any change to a control strategy element that could impact clinically relevant CQAs
*In vivo* bioequivalence documentation	Highly prescriptive based on level of change	Customized based on risk assessment and product performance sensitivities. May need to consider the impact of any change to a control strategy element that could impact clinically relevant CQAs

*PAT* process analytical technologies, *QBD* quality by design, *CQA* critical quality attributes, *QRM* quality risk management

Table I provides a comparison of the current SUPAC IR/MR requirements and the proposed requirements that result when an IR or MR product has been developed using QbD. Included in the table are the following SUPAC change control concepts:

- Types of changes
- Risk evaluation
- Filing documentation
- Manufacturing documentation
- Application of compendial release documentation
- Stability documentation
- Dissolution documentation
- *In vivo* bioequivalence documentation

The following examples provide further detail on the information found in Table I.

1. SUPAC IR Level 1—Batch size change <10× biobatch (final blend size)
*Current SUPAC Requirements*: Annual notification with updated batch records, one lot on long-term stability; release via compendial/application specifications
*Proposed QbD Approach*: No notification if within approved blending design spaces or uniformity is controlled with in-line NIR; documentation managed internally through PQS, including documented risk assessment and justification for stability, extended testing beyond release, and continuous process verification plan
*Rationale*: No stability requirement since no expected change to stability indicating CQA; plan to monitor stability indicating CQA (moisture) and content uniformity using NIR (if applicable). No additional dissolution testing required based on risk assessment. Default to SUPAC requirements if no adequate technical justification can be developed, for example, no stability indicating CQA can be documented in risk assessment
2. SUPAC IR Level 2—Batch size change >10× biobatch (final blend size)
*Current SUPAC Requirements*: Changes being effected supplement with updated batch records; one batch with 3 months’ accelerated stability data and one batch on long-term stability; multipoint dissolution profile comparison in the application/compendial medium *versus* pre-change/reference lot
*Proposed QbD Approach*: No notification if within approved blending design spaces or uniformity is controlled with scale-independent approach such as in-line NIR; documentation managed internally through PQS, including documented risk assessment and justification for stability, extended testing beyond release, and continuous process verification plan
*Rationale*: No stability requirement since no expected change to stability indicating CQA; plan to monitor stability indicating CQA (e.g., moisture) and content uniformity using NIR. No additional dissolution testing required above release with approved clinically relevant dissolution method and specifications or if process remains within the blending design space. Default to SUPAC requirements if no adequate technical justification can be developed, for example, no approved blending design spaces or scale-independent control strategy can be documented in risk assessment
3. SUPAC IR Level 2—Change in granulation process, e.g., change in roller compaction roll pressure set point outside of filed/validated range
*Current SUPAC Requirements*: Changes being effected supplement with updated batch records; one batch on long-term stability; multipoint dissolution profile comparison in the application/compendial medium *versus* pre-change/reference lot
*Proposed QbD Approach*: No notification if within approved roller compaction design spaces or if ribbon attributes controlled within approved in-process controls, for example, using a validated and approved at-line or online ribbon density PAT measurement technique, e.g., NIR or GeoPyc; SUPAC documentation managed internally through PQS, including documented risk assessment and justification for stability, extended testing beyond release, and continuous process verification plan
*Rationale*: No stability requirement since no expected change to stability indicating CQAs or technical justification of no stability impact with change in ribbon density within design space; plan to monitor downstream controls (e.g., tablet hardness and weight uniformity) and ribbon density within design space in addition to the continuous process verification plan for monitoring CQAs. No additional dissolution testing required above release with approved clinically relevant dissolution method and specifications or if process remains within the roller compaction design space. Default to SUPAC requirements if no adequate technical justification can be developed, e.g., outside of approved roller compaction design spaces or no ribbon attribute-based control can be documented in risk assessment
4. SUPAC MR Level 2—Change in coating quantity of rate controlling excipient (ER membrane coating) between 5% and 10% (*w*/*w*) of the tablet mass
*Current SUPAC Requirements*: Prior approval supplement (all information including accelerated stability data) and updated executed batch records; stability for non-narrow therapeutic range drugs: one batch with 3 months’ accelerated stability data reported in prior approval supplement and long-term stability data of first production batch reported in annual report; extended-release dissolution requirement: in addition to application compendial release requirements, multipoint dissolution profiles should be obtained in three other media, for example, in water, 0.1 N HCl, and USP buffer media at pH 4.5 and 6.8 for the changed drug product and the biobatch or marketed batch (unchanged drug product) using appropriate statistical comparison, e.g., *f*_2_ similarity
*Current QbD Approach*: No notification if change is within membrane quantity design spaces or if quantity is controlled within approved in-process controls using a validated and approved at-line or online PAT measurement technique, for example, NIR, Raman spectroscopic techniques; documentation managed internally through PQS, including documented risk assessment and justification for stability, extended testing beyond release, and continuous process verification plan
*Rationale*: No stability requirement since no expected change to stability indicating CQA or technical justification of no stability impact with changes in coating quantity within the approved design space; plan to monitor critical coating parameters and film thickness/quantity within design space in addition to the continuous process verification plan for monitoring CQAs. No additional dissolution testing required above release with approved clinically relevant dissolution method and specifications or if process remains within the coating quantity design space. Default to SUPAC requirements if no adequate technical justification can be developed, for example, outside of approved coating quantity design spaces or insufficient coating quantity in-process control with clinically relevant dissolution assessment that can be documented in risk assessment
5. SUPAC MR Level 3—Change in site of manufacture
*Current SUPAC Requirements*: Prior approval supplement (all information including accelerated stability data) and updated executed batch records; stability with significant body of information not available: three batches with 3 months’ accelerated stability data reported in prior approval supplement and long-term stability data of first three production batches reported in annual report; extended-release dissolution requirement: in addition to application compendial release requirements, multipoint dissolution profile for the changed drug product and the biobatch or marketed batch (unchanged drug product) using appropriate statistical comparison, e.g., *f*_2_ similarity. Bioequivalence documentation: a single-dose bioequivalence study. The bioequivalence study may be waived in the presence of an established IVIVC.
*Proposed QbD Approach*: Notify via Changes Being Effected Supplement (CBE-30) with no changes to approved design spaces and control strategy compared to the original approved site; documentation managed internally through PQS, including documented risk assessment and justification for stability, extended testing beyond release, and continuous process verification plan. Emphasis should be on demonstrating equivalent quality and performance in the supplement through direct confirmation of design spaces at the new site, with additional emphasis on demonstrating equivalent in-process controls and attributes from the approved in-process tests using the at-line or in-line PAT tests (if applicable).
*Rationale*: Consider the change in location of an extended-release coating process to another site. All design spaces were confirmed at the new site without changes to the approved control strategy. From the risk assessment, it was determined that there was no stability CQAs for the drug product that could be monitored or which could be predicted. Therefore, the first three process qualification lots are put on accelerated stability and 3-month data included in the supplement. However, it was demonstrated that control of the controlled-release membrane was found to be equivalent via the approved in-process PAT methods for the controlled-release attribute, film thickness, which was previously shown in development to be clinically relevant and which correlated with dissolution. Consequently, there were no demonstrated changes to the rate-controlling step and, therefore, no bioequivalence study is required. No additional dissolution testing required above release with approved clinically relevant dissolution method and specifications or if process remains within all design spaces. The continuous verification plan would monitor CQAs as well as any in-process controls for the rate-controlling step. Default to SUPAC requirements if all design spaces cannot be confirmed without changes to the approved control strategy from the original site.

#### Improvement in the Control and Characterization of APIs (Analysis and Recommendations Based on Post-approval Change Guidance Documents)

##### Evolution of Drug Substance Control Strategy

Before the introduction of QbD concepts to pharmaceutical manufacturing, the approach to quality control was to test the drug substance to certain predetermined standards. Product quality was assured by ensuring conformance to current good manufacturing practices (GMPs) and end product testing. It needs to be noted that these principles still remain the cornerstone to the quality standards of today and continue to be valid.

ICH has developed guidelines that seek to redefine the traditional approach to ensuring quality by introducing quality-by-design principles. Readers are encouraged to familiarize themselves with the principles of ICH Q8, Q9, Q10, and Q11 in order to gain the needed perspective on development practices.

##### Drug Substance Quality as a Function of Drug Product

Drug product quality and performance considerations are very important to consider in determining the intended quality of drug substance. QTPPs and CQAs of the drug product (as defined in ICH Q8) and previous experience from related products can be helpful in identifying potential CQAs of the drug substance. Quality risk management (QRM; ICH Q9) and knowledge management (ICH Q10) can be used to facilitate manufacturing process development and design of the manufacturing process.

##### Quality by Design: Beginning with the End in Mind…and Working Backwards

QbD principles ensure quality through the entire product life cycle and encompass manufacturing changes both pre- and post-approval. API processes developed through product and process knowledge can result in well-defined and understood material attributes, quality attributes, process parameters, and control strategies that assure product quality throughout the product life cycle.

- Less reliance on end product testing to assure quality
- Efficient use of supply chain infrastructure, thereby enhancing one’s ability to expeditiously respond to changes in product demand
- Better innovation and continuous improvement
- Opportunities for post-approval regulatory flexibility by demonstration of product quality within the proposed design space

The ICH Q11 guidance document (12) describes two different approaches to developing a drug substance: “traditional” and “enhanced.” A sponsor may choose either approach to drug substance development or a combination of both.

In the “traditional” approach, set points and operating ranges for process parameters are defined and the drug substance control strategy is based on the demonstration of process reproducibility and testing to meet established acceptance criteria. NORs and PARs for process parameters may be established under the traditional approach by use of univariate design/analysis of process attributes.

In the “enhanced” approach, risk management principles and scientific knowledge are used more extensively to identify and understand process parameters and unit operations that impact the CQAs and develop appropriate control strategies that are applicable over the life cycle of the drug substance, which may include the establishment of a “design space.”

Design space is submitted to the FDA as part of the marketing application and subject to approval by the agency. In principle, any movement within the approved design space does not constitute a change and, hence, does not need to be reported to the FDA. Conversely, changes that are outside of the approved design space have to be submitted to the FDA for review and approval before the product is distributed. Regulatory flexibility is realized by ensuring that the process operates within the design space, as approved in the original application. The QbD approach enunciates six principal steps in development that should be done in the following order:

- Develop the quality target product profile
- Identify critical quality attributes
- Perform a risk assessment
- Develop design space
- Define control strategy
- Continuous improvement/life cycle management

A well-understood manufacturing process/product performance with a robust control strategy and a nimble manufacturing and supply chain infrastructure that can accommodate varying product demand during development through post-approval while at the same time ensuring lean, just-in-time manufacturing are among the desired attributes of a successful product.

##### Factors Impacting Drug Substance Quality—CPPs and CQAs

A CQA for the drug substance is a physical, chemical, biological, or microbiological property or characteristic that should be within an appropriate limit, range, or distribution to ensure the desired product quality. Drug substance CQAs typically include attributes that affect the identity, purity, biological activity, and stability. Potential CQAs for the drug substance include chemical purity, enantiomeric purity (if applicable), impurities including genotoxic impurities, polymorphic form, particle size, and other attributes that have a direct relationship to the manufacturability, quality, and performance of the drug product. The list of CQAs can be modified as the knowledge of the drug substance and the manufacturing process increase during development

There are many factors that impact the quality of the drug substance and hence need to be part of the overall control strategy, including but not limited to

- Manufacturing site
- Material attributes (e.g., of starting materials, raw materials, reagents, solvents, intermediates, process aids, etc.).
- Manufacturing process including in-process controls and process parameters (e.g., route of synthesis, order of addition of reagents, etc.).
- Analytical methodologies used in the testing of materials (raw materials, starting materials, intermediates, solvents, etc.) and final drug substance
- Drug substance specification (release testing)
- Drug substance container-closure system

**Assessment of CQAs and Continuum of Criticality Throughout the API Product Life Cycle**: Scientific knowledge based on development experience and first principles and QRM techniques are used to reach a decision on the CPPs and CQAs for a given drug substance manufacturing process. In this context, it is important to understand the relationship between risk and criticality of quality attributes and process parameters (10). The criticality of a quality attribute is primarily based upon the severity of harm and does not change as a result of risk management. Criticality of a process parameter is linked to the parameter’s effect on any critical quality attribute. It is based on the probability of occurrence and detectability and therefore can change as a result of risk management.

**Fig. 3:**
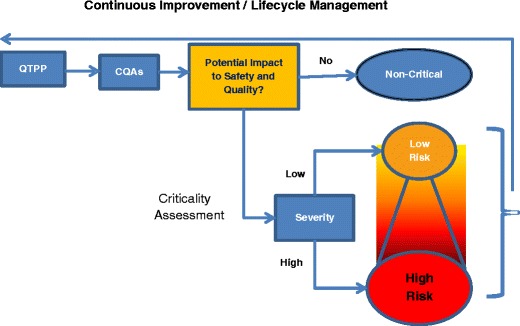
Assessment of the impact of API CQAs on product quality

The drug substance control strategy can be determined by either a traditional approach based on material specifications and process parameter ranges or using an enhanced approach via risk management and scientific knowledge. The iterative nature of product and process development requires that attention is given as early as possible in development, to refine the QTPP, the initial list of potential CQAs, and to move toward the proposed list of CQAs and establishing acceptance criteria for the critical material attributes. An effective control strategy for the drug substance that links changes in drug substance attributes to finished product safety and efficacy ensures that the API manufacturing process consistently produces a product that meets those QTPP and CQAs throughout its life cycle, as shown in Fig. 3 (24).

Design space is typically developed at a small scale and hence has to be supplemented by an effective control strategy to manage the risk after development and implementation.

Development of the design space greatly relies on existing body of knowledge on the drug substance and its manufacturing process. The level of variability present in the historical data will influence the ability to develop a design space, and additional studies should be considered in such cases. A design space may also be determined per unit operation (e.g., crystallization, reaction, distillation, filtration, etc.) or a combination of selected unit operations. The unit operations included in such a design space should be selected based on their impact on the drug substance CQAs.

**Drug Substance Control Strategy**: A control strategy is a planned set of controls, derived from current product and process understanding that assures process performance and product quality (9). The control strategy for API can include any or all of the following:

- Controls on material attributes
- Manufacturing process controls including unit operations (order of addition of reagents, rate of addition, temperature, agitation speed, etc.)
- In-process controls
- Final release testing of the drug substance

The control strategy should ensure that each of the drug substance CQAs is within the appropriate range, limit, or distribution to assure quality of the drug substance that meets all the performance objectives for the drug product. The drug substance specification is only one part of a total control strategy.

The control strategy is typically developed during the clinical research stage of development and undergoes further refinement as further manufacturing experience is gained during clinical development through commercialization. It should be continually evaluated as development progresses and/or when changes are made. The complexity of the process; changes in scale (scale-up or scale-down); differences in the manufacturing process including equipment, facilities, sites, etc.; and change in raw material attributes due to supplier changes, etc., are some important considerations in ongoing evaluations of the drug substance control strategy.

##### Advancements in Instruments and Methods Used in Control and Characterization of Active Pharmaceutical Ingredients—PAT

PAT is a system for designing, analyzing, and controlling manufacturing outputs through timely measurements (i.e., during processing) of critical quality and performance attributes of raw and in-process materials and processes with the goal of ensuring final product quality (25,26). Rapid advances in PAT tools have brought about paradigm shifts in traditional methods and approaches to ensuring drug substance quality.

The underlying principle behind PAT is the belief that quality cannot be tested into the products; it should be built-in or should be by design.

**Drug Substance Process Understanding**: Structured product and process development on a small scale, using experimental design and use of online or in-line instruments to collect data in real time, can provide increased insight and understanding for process development, optimization, scale-up, technology transfer, and control. Process understanding then continues in the production phase when other variables (e.g., environmental and supplier changes) may possibly be encountered. A manufacturing process is generally considered well understood when

- All critical sources of variability are identified and explained.
- The variability is managed by the process controls.
- API product quality attributes can be accurately and reliably predicted over the design space established for materials used, process parameters, manufacturing, environmental, and other conditions.

Focus on process understanding can reduce the burden for re-validation of systems by providing more options for justifying and qualifying systems intended to monitor and control the biological, physical, and/or chemical attributes of materials and processes.

**Methods and Tools**: The last three decades have seen significant progress with respect to methods and tools for chemical attributes (e.g., identity and purity); however, methods and tools to characterize certain physical and mechanical attributes of pharmaceutical ingredients are not necessarily well understood. As a consequence, inherent, undetected variability of raw materials may be manifested in the final product.

Establishing effective processes for managing physical attributes of raw and in-process materials requires a fundamental understanding of attributes that are critical to product quality. Such attributes (e.g., particle size and shape variations within a sample) of raw and in-process materials may pose significant challenges because of inherent complexities related to collecting representative samples.

Many pharmaceutical processes are based on time-defined end points based on prior experience and knowledge derived from process development. However, differences in material attributes and other process conditions can result in variability in API quality attributes that may not necessarily correlate well with the aforementioned time-defined end points. Use of PAT tools and principles can provide valuable, real-time information on process performance and physical, chemical, and biological attributes.

##### Improvements in the Control and Characterization of Critical Excipients

For many years, the impact of excipients, or more specifically the impact of variability in excipient properties, on the CQAs of pharmaceutical products was treated as an uncontrolled, poorly understood variable input to the manufacturing process. Testing of excipients has traditionally focused on pharmacopeial requirements, which are more identity- and purity-centric rather than functionality-oriented. Manufacturing difficulties encountered by various companies over the last 20+ years, especially in compression and lubrication steps, raised the need to characterize the potential impact of excipient variability (i.e., CMA) on CQAs. USP Excipient performance Chapter <1059> has now been established to address additional functional tests (particle size, size distribution, surface area, etc.) which are not covered by individual excipient monographs. The European Pharmacopeial Commission has taken a similar (yet different) approach by adding functionally related characteristics (FRCs) to Ph. Eur. monographs.

Quality-related developments such as GMPs for the twenty-first century (28), the advent of PAT, improved analytical methodology, and the various statistical tools readily available enable better quantification of the impact of variability in excipient lots and parameters on the CQAs. The outcome is that the product is better quantified and, consequently, its risk better managed. Thus, the result will be a more robust manufacturing process and more consistent quality of the finished product.

**Critical Excipients Based on Function**: Excipients are selected to serve particular functions in a product and, therefore, bear the potential to impact either CQA (such as the release rate, uniformity, stability) and/or manufacturability of the drug product. Consequently, any assessment of whether an excipient is critical or not needs to be performed to address its potential impact on either CQA of the product or associated manufacturing process. The identification of which CMAs have the most effect on the CQAs of the drug product should be a risk-based process, in line with ICH Q8(R2). This can be part of a larger study including process parameters with the goal of defining a design space, but the use of a formal design space strategy is not a requirement for assessing the potential impact of material attributes so long as it is done in a systematic manner.

The proposed standard should be a systematic assessment approach starting with a high-level risk assessment, followed by experimental evaluation, and, finally, identification of PARs for CMAs, as outlined below.

- A risk assessment
- Experimental evaluation
- Determination of PAR for critical material attributes
- Confirmation studies and/or real-life production experience

**Risk Assessment**: It is recommended that the evaluation process begin with an initial wide-scope risk assessment. It should be recognized that every component of the unit formula that can be assigned a function is important. All components have some inherent variability. Any component variability that causes a quality attribute to go outside of acceptable ranges is demonstrating a CQA that needs to be carefully defined and controlled. One option for documenting this assessment is via a cause-and-effect matrix. The output of the risk assessment will be a ranking of the potential of each of the excipient material attributes to impact each CQA of the product. It is recommended that this ranking be used as the basis for any experimental evaluation that is conducted.

**Experimental Evaluation**: The purpose of the experimental evaluation is to characterize the impact of known material attribute variability on the CQA of the product and manufacturing process. As mentioned previously, this could be combined with an evaluation of composition and process parameters or performed separately. The risk assessment provides a starting point for determining what should be evaluated, with the focus on the material attributes ranked as having the highest potential to impact the product. There may be practical reasons why some of the material attributes which ranked high cannot be evaluated. For example, all of the lots of material available may have a material attribute within a narrow range even though the allowable range of variation may be broader. Taking practical considerations into account, it is recommended that studies be conducted, preferably using a statistical experimental design, in order to identify the impact of variation in the material attributes. The ranges evaluated for material attributes should take into account the range typically achieved by the manufacturer and how that compares to the excipient manufacturer’s specification, and the manufacturer’s process capability if that is known. For example, in the evaluation of controlled-release matrix formers, it is typical to include viscosity (molecular weight) as a parameter in the design. Given the improvement in computation tools, it is also possible to conduct evaluation using modeling techniques, thereby significantly reducing experimental workload.

**Determination of PAR for Critical Material Attributes**: The appropriate use of statistical experimental designs to assess the impact of material attributes on the CQA of the product, ideally at the same time as assessing processing variables, will result in an understanding of the ranges for these material attributes which will give an acceptable product. This will form part of the design space for the product. In many cases, the design space will be complex and reflect the interactive relationship between variability in material attributes and the variables in the manufacturing process. It is common for sponsors to further implement a tighter NOR based on the established design space in order to enhance process capability.

Many excipients are used to manufacture multiple products. Therefore, the specifications used to identify their fit for purpose need to be constructed based on the complete range of products for which an excipient is used. One option to control excipients for a particular product, when needed, is by the means of establishing additional critical material attributes under a separate internal product code specific to that product.

**Confirmation Studies and Real-Life Production Experience**: Ultimately, designation of critical material attributes and process parameters will either be proven or disproven through additional confirmation studies involving multi-sourced excipients. Vendor-to-vendor excipient differences will be highlighted, if any, through excipient qualification studies and, when warranted, confirmation batches of the drug product. As routine production commences using more excipient batches, real-life data can be evaluated to confirm selected NOR or revised accordingly in the spirit of continued process verification, as outlined in the FDA guidance on process validation issued in January 2011 (27,28).

**Additional Considerations Other Than Functionality**: Although excipients are selected based on their intended functionality, many times, minor impurities associated with excipients bring unanticipated challenges to a drug product’s long-term performance. It is common knowledge that peroxides, anhydrides, and other organic solvents associated with excipients can interact with the API, forming degradation products, thereby causing stability concerns. These impurities and residual solvents can be either inherent from the excipient source (i.e., natural products), formed during the excipient manufacturing process, or liberated from the excipient during the drug product manufacturing process. Regardless, vigilance should be paid to the evaluation of such excipient impurities or residual solvents as part of stressed manufacturing process studies (e.g., known susceptibility at extremes of temperature or force) or stressed stability conditions. If needed, control of the levels of these impurities or residual solvents would warrant incoming specifications to assure long-term drug product performance.

### Role of IVIVC in the Development and Scale-Up of IR and MR Products

The term *in vitro*–*in vivo* correlation (IVIVC) refers to the establishment of a rational relationship between an *in vivo* parameter derived from drug plasma concentrations produced by a dosage form (e.g., *C*_max_ or AUC) and an *in vitro* characteristic of the same dosage form (e.g., dissolution) (29). The quantitative relationship between the *in vivo* and *in vitro* properties is an IVIVC. The most important utilization of an IVIVC is that of predictability of the clinical performance of a dosage form based on dissolution data. In some cases, the actual drug plasma concentration profile may be predicted from *in vitro* dissolution data. If prediction cannot be accomplished, it does not mean that the *in vitro* method is necessarily invalid. If a relationship cannot be established between the dissolution and bioavailability of a dosage form, an *in vitro*–*in vivo* “relationship” or “association” can still be of great value to a formulation group when the dissolution method is predictive of *in vivo* behavior.

Historically, IVIVC analysis has been more successful for modified-release products than for immediate-release dosage forms. This difference in success probably reflects the application of specific data analysis techniques and interpretation that require dissolution rate-limited drug absorption and that the release of drug from extended-release dosage forms can be significantly affected by the drug product formulation. Numerous attempts have been made to correlate various *in vivo* PK parameters with *in vitro* dissolution data. Single-point correlations show that increasing or decreasing the *in vitro* dissolution rate of a modified-release dosage form can result in a corresponding directional change in the *in vivo* performance of the product (e.g., *C*_max_ or AUC increase with an increase in the dissolution rate). However, such single-point correlations do not reveal much information regarding the overall plasma concentration–time profile. Thus, correlation methods that utilize all available plasma drug concentration and *in vitro* data are preferred. There are three correlation levels available that utilize dissolution and plasma data, but there are significant differences in the quality of the correlation obtained with each procedure. These methods or correlation levels are outlined below in terms of the advantages of each along with its potential utility as a predictive tool.

Three correlation levels have been defined and categorized in descending order of the quality of predictability (29–31). The concept of correlation level is based upon the ability of the correlation to reflect the entire plasma drug concentration–time curve that will result from administration of the given dosage form. It also relates the entire *in vitro* dissolution curve to the entire plasma concentration–time profile; the strength of this relationship defines its inherent predictability.

#### Level A Correlation

Level A correlation is the highest category of correlation. It represents a point-to-point relationship between the *in vivo* input rate (absorption rate) and *in vitro* dissolution of the drug. For a level A correlation, a product’s *in vitro* dissolution curve is compared to its *in vivo* input curve. This *in vivo* curve, produced by deconvolution of the plasma profile, may be accomplished using mass balance model-dependent techniques, such as the Wagner–Nelson or Loo–Riegelman methods, or by model-independent mathematical deconvolution. Ideally (though not an absolute requirement), the *in vitro* and *in vivo* curves are superimposable or may be made to be superimposed by the use of a constant offset value of the timescale, and therefore the equations describing each curve are the same. Such a relationship is most often found with those modified-release dosage systems that demonstrate an *in vitro* release rate that is independent of the dissolution media and stirring speeds employed in a dissolution apparatus. The advantages of a level A correlation are as follows:

- A point-to-point correlation utilizes every plasma level and dissolution point collected at different time intervals and, thus, reflects the complete plasma level curve. With a level A correlation, the *in vitro* dissolution curve may serve as a surrogate for *in vivo* performance. Therefore, changes in manufacturing site, method of manufacture, raw materials, minor formulation modifications, and even product strength using the same formulation can be justified without the need for additional bioavailability/bioequivalence studies so long as the product’s dissolution profile is unaffected.
- The *in vitro* dissolution method is validated as a quality control procedure that is meaningful and predictive of a dosage form’s *in vivo* performance.
- The extremes of the *in vitro* quality control standards for a product (e.g., TPP) can be justified either by convolution (simulating the plasma level profile from the dissolution curve) or by a deconvolution procedure (utilizing the upper and lower confidence interval limits).

#### Level B Correlation

This correlation utilizes the principles of statistical moment analysis. The mean *in vitro* dissolution time is compared to either the mean residence time or the mean *in vivo* dissolution time. As with a level A correlation, level B utilizes all of the *in vitro* and *in vivo* data, but is not a point-to-point correlation. It does not correlate the actual *in vivo* plasma profiles, but rather a parameter that results from statistical moment analysis of the plasma profile such as mean residence time. Because there are a number of different plasma profiles (shapes) that will produce similar mean residence time values, it is not possible to rely upon a level B correlation alone to predict a plasma profile from *in vitro* dissolution data. Also, *in vitro* data from a level B correlation cannot be used to justify the extremes of a product’s quality control standards.

#### Level C Correlation

This category relates one dissolution time point (*t*_50%_, *t*_90%_, etc.) to a single pharmacokinetic parameter such as AUC, *C*_max_, or *T*_max_. Similar to a level B correlation, a level C correlation represents a single-point correlation and does not reflect the complete shape of the plasma profile which best defines the performance of modified-release products. Since this type of correlation is not predictive of actual *in vivo* product performance, it is generally only useful as a guide in formulation development or as a production quality control procedure. Because of its obvious limitations, a level C correlation has limited usefulness in predicting *in vivo* drug performance and is subject to the same caveats as a level B correlation in its ability to support product and site changes as well as justification of quality control standard extremes.

#### Correlation for IR Dosage Forms

Since the mechanisms for release of drug from immediate-release dosage forms are simpler than that for modified-release dosage forms (which are more complex and variable), one might expect that an IVIVC would be easier to develop with IR formulations. However, most of the correlation efforts to date with immediate-release dosage forms have been based on the correlation level C approach, although there also have been efforts employing statistical moment theory (level B). Although it is conceivable that the same level A correlation approach may be utilized with immediate-release dosage forms, until data have been gathered to support this concept, levels B and C are the best approaches that can be recommended with these dosage forms.

#### General Considerations for Developing IVIVCs

The following concepts were adopted from the FDA IVIVC Guidance (29). General considerations for developing and evaluating a level A *in vitro*–*in vivo* correlation include

- Human data should be used for regulatory consideration.
- IVIVCs should utilize sufficient subjects (a statistically powered sample) to adequately characterize the drug product performance. Crossover studies are preferred, but appropriately powered parallel studies may be acceptable.
- It is expected that, in order to develop a correlation between a drug product’s PK and its dissolution, several formulations with different release rates may need to be evaluated. These formulations should have at least a 10% difference in dissolution rates.
- The IVIVC should be demonstrated consistently, with two or more formulations with different release rates that result in corresponding differences in absorption profiles. Although an IVIVC can be defined with a minimum of two formulations with different release rates, three or more formulations with different release rates are recommended in order to establish a more robust relationship.
- Predictability of the IVIVC should be assessed. An average absolute percent prediction error of 110% or less for *C*_max_ and AUC is preferred.

#### Use of IVIVC in Post-approval Change Justification

##### Use of Biowaiver Options in Post-approval Changes for IR and MR Products

The following section on biowaivers is written based on the current US FDA guidance(s) and pertinent CFRs. The Biopharmaceutical Classification System (BCS) and other related guidances are in the process of being (or soon will be) updated; therefore, guidelines to request biowaivers may change accordingly in the future.

##### Regulatory Applications of Biowaivers

In many cases, *in vivo* bioequivalence studies can be replaced by *in vitro* dissolution studies in regulatory applications. The application can be based on BCS or IVIVC biowaivers or simply on a dissolution profile comparison. Both BCS and IVIVC biowaivers can be utilized to support post-approval changes in drug product composition, manufacturing site, or production method.

Approval of new, lower strengths or some minor changes to the drug product can sometimes be applied for and justified based solely on the dissolution profile comparison without the need for IVIVC or BCS-based biowaivers. In such cases, in order to assure bioequivalence, the new dose strength or new formulation should have a similar dissolution profile to that of the drug formulation already on the market. Examples of biowaiver applications are

- Biowaivers for comparative bioavailability studies in support of a New Drug Application
- Biowaivers for comparative bioavailability studies in support of the bioequivalence of subsequent-entry products to the US market
- Biowaivers for bridging studies where the formulation to be marketed is different from the formulation used in the pivotal clinical trials
- Biowaivers for studies in support of significant post-approval changes and product line extensions

##### General Considerations

Waiver of *in vivo* studies for different strengths of a drug product can be granted under Section 320.22(d)(2) when the following requirements are meet: (1) the drug product is in the same dosage form, but in a different strength; (2) this different strength is proportionally similar in its active and inactive ingredients to the strength of the product for which the same manufacturer has conducted an appropriate *in vivo* study; (3) there is evidence of linear kinetics in the proposed dosing range; and (4) the new strength meets appropriate *in vitro* dissolution and similarity *f*_2_ tests.

The “proportionally similar” requirement is defined in the following ways:

- All active and inactive ingredients are in exactly the same proportion between different strengths (e.g., a tablet of 50-mg strength has all the inactive ingredients, exactly half that of a tablet of 100-mg strength, and twice that of a tablet of 25-mg strength).
- Active and inactive ingredients are not in exactly the same proportion between different strengths as stated above, but the ratios of inactive ingredients to the total weight of the dosage form are within the limits defined by the SUPAC-IR and SUPAC-MR guidances up to and including level II.
- For drug products, where the amount of the active drug substance in the dosage form is relatively low compared to the excipients, the total weight of the dosage form remains nearly the same for all strengths (within +10% of the total weight of the strength on which a bio-study was performed), the same inactive ingredients are used for all strengths, and the change in any strength is obtained by altering the amount of the active ingredients and one or more of the inactive ingredients. The changes in the inactive ingredients are within the limits defined by the SUPAC-IR and SUPAC-MR guidances up to and including level II.
- In interpreting the guidance, it is important to note that combination products (i.e., bilayer tablets, etc.) are considered to be one formulation even though they consist of two separate layers with different formulations. In assessing the proportional similarity of the different strengths, all components of both layers have to be proportionally similar. The fact that only one layer is proportionally similar and the other is not clearly indicates that the product (whole tablet) is not proportionally similar. In many instances, there might be interactions among the different tablet layers that might be different across different strengths due to the different sizes of the layers and the varying amounts of excipients present in each layer.
- Exceptions to the above definitions may be possible, if adequate justification is provided.

Biowaivers for IR and MR solid oral products can be classified under three major categories, as described in the following section:

##### Biowaivers Without n IVIVC

**Immediate-Release Formulations (Capsules, Tablets, and Suspensions)**: Dissolution profiles should be generated for all strengths. If an appropriate dissolution method has been established and the dissolution results indicate that the dissolution characteristics of the product are not dependent on the pH and product strength, then dissolution profiles in one medium are usually sufficient to support waivers of *in vivo* testing. Otherwise, dissolution data in at least three media (e.g., pH 1.2, 4.5, and 6.8) are needed. The *f*_2_ test should be used to compare profiles from the different strengths of the product. An *f*_2_ value >50 indicates a sufficiently similar dissolution profile such that further *in vivo* studies are not needed. For an *f*_2_ value <50, further discussions with the appropriate review division may help determine whether an *in vivo* study is needed. The *f*_2_ approach is not suitable for rapidly dissolving drug products (e.g., >85% dissolved in 15 min or less).

**Biowaivers for Lower-/Higher-Strength Products**: When several strengths of the same dosage form are proportionally similar in their active and inactive ingredients, an *in vivo* waiver can be granted for the lower strength(s) based on comparability of the dissolution profiles and an acceptable *in vivo* BA or BE study on the highest strength. Similarly, it is also possible to get a waiver for a higher strength based on the assessment of (1) clinical safety and/or efficacy data on the proposed dose and the need for the higher strength, (2) linearity of the pharmacokinetics over the therapeutic dose range, (3) the higher strength is compositionally proportionally similar to the lower strength,^1^ and (4) the same dissolution procedures being used for all strengths and similar dissolution results are obtained.

**Biowaivers for Over-encapsulation of Clinical Trial Formulations**: During the course of drug development, sponsors sometimes have to blind the formulations that they use in the clinical trials. In certain situations, the only difference between the market and clinical trial formulation is that the tablet mix or the tablet itself is put into a capsule. This is done mainly for blinding purposes. It is thus possible to obtain a waiver for the bioequivalence study that links the market and clinical trial formulation provided that no other excipients are added to the capsule that are known to affect the release of the active drug from the capsule. The waiver of this *in vivo* bioequivalence study is granted based on the comparability of the dissolution profile in three media: 0.1 N HCl and phosphate buffers pH 4.5 and 6.8.

**Biowaivers During Scale-Up and Post-approval Changes**: Certain formulation changes in components and composition, scale-up, manufacturing site change, manufacturing process, or equipment changes may be made post-approval. Depending on the possible impact of the manufacturing change on the release of the active ingredient and its bioavailability from that formulation, certain manufacturing changes for IR products can be approved solely based on comparability of the dissolution profiles between the post-change and pre-change formulations. Information on biowaivers (and the requirements of *in vitro* dissolution and *in vivo* BE studies) for immediate-release drug products is provided in the FDA’s SUPAC IR Guidance for Industry: Immediate-Release Solid Oral Dosage Forms: Scale-Up and Post-approval Changes: Chemistry, Manufacturing, and Controls, *In Vitro* Dissolution Testing, and *In Vivo* Bioequivalence Documentation (31).

**Biowaivers for Modified-Release Formulations**: Information on alternative methods to demonstrate bioequivalence for modified-release drug products approved in the presence of specified post-approval changes is provided in an FDA guidance for industry entitled SUPAC-MR: Modified-Release Solid Oral Dosage Forms: Scale-Up and Post-approval Changes: Chemistry, Manufacturing, and Controls, *In Vitro* Dissolution Testing, and *In Vivo* Bioequivalence Documentation (32). The same principles described in the guidance may be applied to pre- and post-approval changes.

**Beaded Capsules: Lower/Higher Strength**: For extended-release beaded capsules where the strength differs only in the number of beads containing the active moiety, a single-dose, fasting PK or BE study, as appropriate, should be carried out on the highest strength, with a request for a waiver of the *in vivo* studies for the lower strengths based on comparative dissolution profiles. The dissolution profile for each strength should be generated using the recommended dissolution method. If the dissolution method has not been finalized, then dissolution profiles should be generated in at least three media (e.g., pH 1.2, 4.5, and 6.8). *In vivo* BE studies for higher strengths may not be necessary based on (1) clinical safety and/or efficacy data on the proposed dose and the need for the higher strength, (2) linearity of pharmacokinetics over the therapeutic dose range, and (3) the same dissolution procedures being used for all strengths and similar dissolution results obtained. The *f*_2_ test can be used to compare profiles for the different strengths of the product. An *f*_2_ value of >50 can be used to confirm that further *in vivo* studies are not needed.

**ER Tablets—Lower Strength**: For extended-release tablets, when the drug product is in the same dosage form but in a different strength and when (1) drug exhibits linear PK, (2) the various strengths are proportionally similar in its active and inactive ingredients,^2^ and (3) has the same drug release mechanism, an *in vivo* BA or BE determination of one or more lower strengths can be demonstrated based on dissolution profile comparisons, with an *in vivo* BA or BE study only on the highest strength. The dissolution profile for each strength should be generated using the recommended dissolution method. If the dissolution method has not been finalized, then dissolution profiles should be generated in at least three media (e.g., pH 1.2, 4.5, and 6.8). The dissolution profile should be generated on the test and reference products of all strengths using the same dissolution test conditions.

##### Biowaivers Based on IVIVC

With a predictive IVIVC, *in vitro* dissolution would not only be a tool to assure the consistent performance of the formulation from lot to lot but would also become a surrogate for the *in vivo* performance of the drug product. The ability to predict the plasma concentration time profile from *in vitro* data will reduce the number of studies required to approve and maintain a drug product on the market, therefore reducing the regulatory burden on the pharmaceutical industry.

Once an IVIVC has been established, it is possible to waive the requirements for bioavailability/bioequivalence studies. For example, a biowaiver can be granted for pre- and post-approval level 3 manufacturing and process changes, approval of lower strengths, or level 3 formulation changes with complete removal or replacement of excipients (both non-release and release-controlling excipients).

If the IVIVC is developed with the highest strength, waivers for changes made with the lowest strengths are possible if these strengths are compositionally proportional or qualitatively the same, the *in vitro* dissolution profiles are similar, and all the strengths have the same release mechanism.

An IVIVC cannot be used to gain the approval of (a) a new formulation with a different release mechanism, (b) a dosage strength higher or lower than the doses that have been shown to be safe and effective in the clinical trials, (c) another sponsor’s oral controlled-release product even with the same release mechanism, and (d) a formulation change involving an excipient that will significantly affect drug absorption.

For modified-release formulations, it is possible to obtain *in vivo* bioavailability/bioequivalence waivers based on *in vitro* dissolution for changes in formulations that usually require an *in vivo* study. The regulatory criteria for granting biowaivers are outlined in the previously cited FDA guidance on this topic. Basically, the mean predicted *C*_max_ and AUC from the respective *in vitro* dissolution profiles should differ from each other by more than 20%.

For information on IVIVC waivers, refer to the FDA Guidance for Industry: Extended-Release Oral Dosage Forms: Development, Evaluation, and Application of *In Vitro*/*In Vivo* Correlations (33).

##### Biowaivers Based on the Biopharmaceutical Classification System

The BCS is a scientific framework for classifying drug substances based on two fundamental properties of a drug substance, i.e., its aqueous solubility and intestinal permeability. A drug substance can have either a high or low aqueous solubility as well as either a high or low intestinal permeability. In addition, the BCS also takes into account the drug product dissolution which can be either rapid or slow. Thus, the BCS takes into account three major factors that govern the rate and extent of drug absorption from IR solid oral dosage forms: dissolution, solubility, and intestinal permeability (including gastric stability).

In the USA, a BCS-based biowaiver is limited only to class 1 (high solubility–high permeability) drug substances. A BCS biowaiver is acceptable for BCS class I drugs formulated as rapidly dissolving immediate-release products. In that case, the application may be based on *in vitro* dissolution and permeability data together with scientific justification of linear pharmacokinetics, a proof that the drug does not have a narrow therapeutic index and that the excipients do not have pharmacokinetic interactions with the drug.

BCS-based biowaivers are meant to reduce the need for establishing *in vivo* bioavailability/bioequivalence in situations where *in vitro* data may be considered to provide a reasonable estimate of the *in vivo* performance of the immediate-release solid oral pharmaceutical drug products. For information on BCS waivers, refer to the FDA Guidance for Industry: Waiver of *In Vivo* Bioavailability and Bioequivalence Studies for Immediate-Release Solid Oral Dosage Forms Based on a Biopharmaceutics Classification System.

The approach outlined in the BCS guidance can be used to justify biowaivers for highly soluble and highly permeable drug substances (i.e., class 1) in IR solid oral dosage forms that exhibit rapid *in vitro* dissolution using the recommended test methods (21 CFR 320.22(e)). A drug substance is considered highly soluble when the highest dose strength is soluble in 250 mL or less of aqueous media over the pH range of 1–7.5. In the absence of evidence suggesting instability in the gastrointestinal tract, a drug substance is considered to be highly permeable when the extent of absorption in humans is determined to be 90% or more of an administered dose based on a mass balance determination or in comparison to an intravenous reference dose. Alternatively, nonhuman systems capable of predicting the extent of drug absorption in humans can be used (e.g., *in vitro* epithelial cell culture methods). In the BCS guidance, an IR drug product is considered rapidly dissolving when no less than 85% of the labeled amount of the drug substance dissolves within 30 min, using USP Apparatus I at 100 rpm (or Apparatus II at 50 rpm) in a volume of 900 mL or less in each of the following media: (1) 0.1 N HCl or simulated gastric fluid USP without enzymes; (2) a pH 4.5 buffer; and (3) a pH 6.8 buffer or simulated intestinal fluid USP without enzymes.

Examples of cases where this guidance applies are

- Biowaivers for comparative bioavailability studies in support of New Drug Application
- Biowaivers for comparative bioavailability studies in support of the bioequivalence of subsequent-entry products to the US market
- Biowaivers for bridging studies where the formulation to be marketed is different from the formulation used in the pivotal clinical trials
- Biowaivers for studies in support of significant post-approval changes and product line extensions

In all these cases, samples should be collected at a sufficient number of intervals to characterize the dissolution profile of the drug product (e.g., 10, 15, 20, and 30 min). When comparing the test and reference products, dissolution profiles should be compared using a similarity factor (*f*_2_). Two dissolution profiles are considered similar when the *f*_2_ value is ≥50. However, when both test and reference products dissolve 85% or more of the label amount of the drug in <15 min using all three dissolution media recommended above, the profile comparison with an *f*_2_ test is unnecessary.

### Improvements in the Control of Product Scale-Up and Validation

#### Introduction

Process validation was often achieved by the statistically unsound practice of demonstrating that three batches could be made, within specifications, at commercial scale and at the commercial manufacturing site. This may or may not have been preceded by a trial batch to demonstrate the ability to scale-up.

Current regulatory authority and industry expectations, however, are for an approach which integrates scale-up and validation with product development and process understanding. This is aligned with the risk-based development and QbD approaches discussed in previous sections of this publication. This can be envisioned as a two-step process. First, use a scientific, statistics-based system to characterize materials, formulations, and processes and the factors which influence the potential of a product to meet CQAs during development; second, connect and utilize this information in the scale-up and validation process.

The concept of integrating development and validation activities is addressed in the EMEA Guidance Document on Process Validation, made effective in September 2001 and brought more up to date with the FDA Guidance document “Process Validation, General Principles and Practices,” issued in January 2011 (34). This document highlights the continuum from development to commercialization and how knowledge gained throughout this process should be used to ensure that the product continues to have the desired CQAs.

#### Integration with the Development Program

The scale-up of the drug product manufacturing process needs to be aligned with the rest of the development program. The mandatory requirement is that scale-up and validation be completed prior to commercialization, but in practice, aspects such as meeting the requirements to supply product for the pivotal clinical program often move the manufacturing process scale-up much earlier in the project timeline.

If the intention is to perform pivotal clinical studies with the to-be-marketed product, then this can provide an anchor point for the scale-up activities around which the rest of the program can be constructed.

There are many good reasons to move scale-up prior to the pivotal studies. It will often make the development program simpler and remove the need for a BE study to link the clinical and commercial formulations; it will also enable the scientists to gain more knowledge of the commercial process than if scale-up were performed at the end of the development process (and provides more time to address any issues identified) and will provide material for pivotal stability studies.

The negative aspects include, in many cases, the scale-up activity being performed in parallel with the phase 2 clinical program, where projects have the highest probability of termination. In addition, other aspects, such as development of the API, may need to be moved forward and the range of doses included in the scale-up program may be wider than those eventually commercialized.

#### Scale-Up

For most drug products, practical reasons dictate that the majority of studies to obtain knowledge about how material attributes and process parameters impact the CQAs of the drug product will be performed at a scale significantly smaller than commercial. However, conclusions relating to acceptable ranges for process parameters and material attributes need to be drawn from these studies. Importantly, the impact of material attribute differences that may result from multiple suppliers needs to be assessed. The impact of scale on acceptable ranges will be specific for each attribute or parameter and needs to be thought through using a combination of acquired product-specific knowledge and information on the operating principles of the equipment.

In order to better address the impact of changes in scale, it is strongly recommended that companies integrate their lab-, pilot-, and production-scale equipment around specific operating principles as much as possible. For example, if the production facility has top-drive granulators, then use similar, but smaller, top-drive granulators in development. Similarly, if the production facility has bin blenders, then use smaller, but equivalent, blenders in development. Introducing a change in equipment type at the transfer to production scale adds an unnecessary level of variability.

The scale-up process should be risk-based and begin with a baseline of product knowledge acquired from prior stages of the development program. It should also begin with the final commercial process in mind—including an estimated commercial scale of manufacture and probable manufacturing site. Potential suppliers of each of the excipients should be identified, with any differences in physical or quality characteristics (e.g., grade, density, particle size, crystal habit) fully evaluated in any assessments of risk, and API representative of that which will be used commercially should be available. If the API development process is not at a stage where API is representative of commercial, then the material available for the scale-up activities will become the target for the commercial process. Whether this is a reasonable risk to take depends on the specific API and drug product.

The general pattern that the scale-up activities will follow should be known prior to conducting the risk assessment. The most typical approach is to perform blocks of studies where the number of experiments and ranges evaluated decrease as the scale increases. Therefore, following a risk assessment, the output will be assessed to determine what can meaningfully be evaluated at a small scale. A large number of lab-scale batches are typically conducted as a series of statistical experimental designs whereby the ranges for parameters are wider than anticipated at commercial scale. If many parameters are being evaluated, these may be simple, two-level screening-type designs. The knowledge obtained on the design space from the lab-scale studies then provides a foundation for a smaller number of batches at a larger (pilot) scale, with fewer parameters and attributes being evaluated and those that are evaluated having narrower ranges. Parameters found not to be critical at lab scale may not be evaluated at pilot or commercial scale. Statistical designs at pilot scale are typically more powerful, enabling a more detailed assessment of the design space to be obtained. Simple designs are adequate to screen and select variables, whereas more powerful designs allow the optimization of these same variables.

The assessment of the data from the pilot-scale studies could include a comparison with the data from lab-scale studies to characterize the impact of scale. There are several potential benefits of this. The most obvious one would be the opportunity to use lab- or pilot-scale experiments, rather than commercial scale, to assess the impact of desired changes to a commercial process.

After an assessment of the data from these pilot-scale studies, the plans for process evaluation at commercial scale are prepared, with fewer parameters being evaluated and narrower ranges than pilot scale. In most cases, limitations such as API availability and cost will restrict evaluations at this scale to a very small number of batches, which may be made at extremes (e.g., corner points of the design space) of the ranges for commercial manufacture. The number of batches possible is unlikely to be large enough for a statistical experimental design. The parameters evaluated should include any parameter thought to be difficult to assess at a scale smaller than commercial.

A clear understanding of how a process changes with scale is critical to being able to conduct the risk assessments and determine which parameters to evaluate at each scale. In some cases, e.g., milling, a result obtained as the acceptable range for a parameter at lab scale may be directly transferable to commercial scale, whereas in others, e.g., granulation, the impact of scale is more complex, and although small-scale studies may show the extent to which a process is robust, direct conclusions regarding process parameters such as impeller speeds are not usually possible. For this reason, continuous unit operations such as dry granulation are gaining in popularity since scale-up is facilitated by the transferability of small-scale data.

Potential interactions between parameters or parameters and material attributes must also be taken into consideration. An example of this which is easy to visualize is the interaction between duration and capacity or duration and material density for a blending process. An experimental design that includes assessment of the interactions between both parameters and material attributes can often uncover important interactions.

Throughout the scale-up process, extensive monitoring of the batches should be performed. This will be process-dependent, but could include blend uniformity testing at multiple blending times, extensive stratified sampling for uniformity during compression or encapsulation, or monitoring of processes in real time using PAT techniques. The focus should be on areas considered to be high potential risks to successful commercial manufacture. This will add to the body of process knowledge available.

#### Validation

The FDA Guidance document “Process Validation—General Principles and Practices” (34) introduced the concept of three stages of validation with an emphasis on acquiring and using process knowledge in each successive step. The three stages are

Stage 1 *Process Design*: The commercial manufacturing process is defined during this stage based on knowledge gained through development and scale-up activities.
  The development and scale-up activities described in earlier sections of this whitepaper address stage 1 requirements.
Stage 2 *Process Qualification*: During this stage, the process design is evaluated to determine whether the process is capable of reproducible commercial manufacturing.
  This is the section which most closely parallels the concept of making three consecutive successful batches at commercial scale. The FDA guidance document (35) refers to the verification that products can be successfully manufactured at commercial scale as Process Performance Qualification and mandates that the decision to begin commercial distribution of a product must be supported by data from commercial-scale batches. The commercial-scale batches included in the scale-up of the manufacturing process could provide the basis for this section, but the number of batches needed should be specific for the product and process and additional batches may be required to verify ranges for example.
Stage 3 *Continued Process Verification*: Ongoing assurance is gained during routine production that the process remains in a state of control.
  The third stage of validation relates to data collection during routine production to continuously monitor the process. The data should be collected and statistically analyzed to look for trends. This database also facilitates problem solving and simplifies trouble shooting. Many companies were routinely doing this prior to the FDA Guidance document in order to determine process capability, anticipate potential problems, and provide a database which could be statistically mined in the event of any process troubleshooting being needed.

### Improvements in Finished Product Testing

#### Real-Time Testing Approaches

The aim of QbD is to make more effective use of innovative science, technology, and engineering principles in order to obtain and maintain process knowledge throughout the life cycle of a product (25).

This knowledge provides the potential for regulatory relief that can be realized starting from initial product approval (i.e., through replacement of batch end product testing with RTRT) as well as continued relief throughout the product’s life cycle (i.e., through flexible introduction of post-approval changes without prior approval). Identification of CPPs coupled with a robust control strategy designed to assure adherence of the design space to its predetermined ranges involves close to real-time process assessment to ensure that the process is operating in conditions to deliver predetermined end product quality, thereby reducing the need, and more importantly the value, of end product testing. Release based on manufacture and control within the design space alone, however, is not sufficient to assure quality of the drug product during the manufacturing process. In order to support RTRT, appropriate surrogates for affected CQAs as a function of quantified in-process parameters and/or material attributes need to be established and included in release specifications (25).

### Improvements in Post-approval Testing Schemes

Critical parameters identified and monitored at pertinent junctures of the process as part of a control strategy allow for a shift in the focus of quality assessment from end product testing to the testing of surrogate process steps, parameters, and inputs further upstream that have been demonstrated to be directly linked with product performance. As previously mentioned, correlation of a CPP with its specific CQA is essential when establishing impact and mitigating risk to DP quality. Once this correlation is determined, optimization experiments can be done to deepen the understanding of the relationship to the point where correlation evolves into causation and, ultimately, prediction of a response with certain factor conditions is possible. Causal relationships between parameters and quality attributes allow for surrogate substitution of release testing with parameter values further upstream of the process. When striving for RTRT, quantification of the release data needs to be replaced with quantification of another parameter/value. For example, traditional dissolution release testing could be replaced by disintegration time determination, if sufficient knowledge is attained and correlation established.

Incoming raw material specifications can be valuable in correlating with final drug product quality attributes. This is especially true when functional excipients are considered. The effect main excipients have on DP quality should be assessed during product development. It is here that the theoretical impact and potential for affect are investigated and demonstration of actual impact determined. This is important because the attributes of the excipient should not be considered alone (i.e., magnesium stearate can affect dissolution). Rather, the technology platform (i.e., dry blend *versus* wet granulation) should be considered along with the drug substance characteristics, level of excipient in the composition, as well as drug load. The collection of physical and chemical effects allow for a robust understanding of material characteristics that do indeed affect ultimate quality. Once established, quantification of the excipient’s specific attribute causing the response can be established. For example, the particle size distribution of a disintegrant may be critical for one drug product due to a relatively high quantity present in the formulation or particle size distribution variability which affects the way in which the excipient behaves during processing. Raw material controls represent one of the earliest intervention and control capabilities in a process.

In-process controls are samples taken at various time points in any unit operation. They are designed to test for a specific attribute of the current operating step. For example, loss on drying (LOD) is a traditional method of establishing how much water is present in a wet granulation and is used to determine the end point of drying processes. As LOD sampling is known for its inherent variability based on procedure, operator, and experience, it highlights that IPCs are only as valuable as the accuracy of the information they assess. Generally, IPCs are designed to evaluate the current unit operation quality. When striving for RTRT, one must consider how a specific IPC affects not only the current unit operation but the ultimate drug product as well. For example, disintegration time (DT) is commonly used in pharmaceutical compression operations to assess hardness characteristics and the likelihood a tablet will dissolve within an appropriate time frame in order to support target dissolution profiles. If it is possible to understand the relationship between DT and dissolution, demonstration can be performed that if the DT specification is met then the resultant dissolution will be acceptable; one can propose that control tablet DT is adequate for assuring acceptable drug release.

As discussed in the previous section, there are many examples of PAT tools used to enhance process knowledge during pharmaceutical development and production. However, when these tools are desired for use as elements of a control strategy, selection of a particular technology as well as appropriate placement of probes at relevant operation steps are decisions that should be made with consideration given to a specific product/process. RTRT strategy involving PAT should be individualized based on adequate level of control, assessment potential with specific CQA for which the technology is a surrogate, and quantification potential that will replace the traditional end product test to demonstrate acceptable process progression and ultimate product quality.

PAT affords innovative methods for retrieving dynamic process data in real time or close to real time. Ideally, when developing a control strategy that involves PAT tools, alignment with critical process parameters as it relates to the direct output of a specific unit operation as well as relation to final product quality should be attained. In short, DoEs establish the correlation between a process parameter and specific quality attributes, and PAT provides the means of detection and/or control of the parameter assuring acceptable quality output. Positioning of PAT tools in-line, at-line, or off-line can also be assessed for their potential in identifying, explaining, and/or managing process variability. For example, PAT tools such as NIR or Raman can be used during hot melt extrusion for assessing polymer layer thickness in multi-polymer systems and the crystal form of the drug (36). When placed online, the data collected can be used for process adjustment upstream (via a feedback loops) or downstream (via feed-forward loops) based upon process knowledge of how the parameter and related output affect the final product quality.

### Developing Improvements that Hold Future Promise

#### Batch *Versus* Continuous Processing

##### Background

Pharmaceutical production can be broken into two possibilities: batch and continuous. Traditionally, pharmaceutical production is batch-oriented. True continuous processing is just being developed to current regulatory standards and is still relatively unknown in pharmaceutical production. Adoption of continuous processing is challenged by traditional production facilities, operational processes, and equipment, but offers a mechanism for rationalizing many current regulatory concepts.

Current pharmaceutical technology is grounded in batch production processes. Fixed amounts of materials are added to large blending equipment and mixed as a batch prior to the next processing step. Production continues in this manner, with the entire batch completing each process step prior to continuing to the next operation. This method generally requires relatively large equipment, large operational facilities, and some risk that any deviation will introduce quality issues to the entire batch.

While current pharmaceutical production is considered a batch process, common production trains are often a mix of batch and semi-continuous operations. Blenders, mixers, and fluid bed dryers are generally designed to contain the entire product lot and are obviously batch operations. Units such as mills, extruders, tablet presses, and fillers can be viewed as continuous or semi-continuous. Testing is commonly performed over the entire batch, as is QA review and release. Quality issues at any part of the batch may result in the entire batch being rejected.

With continuous processing, material is constantly added and removed from the process train. Multiple activities (e.g., dry blending, wetting, granulation, extrusion) can occur simultaneously inside a single piece of equipment. Individual continuous processing equipment generally has a smaller footprint than its batch equivalent and only contains a small fraction of the product “batch” at any given time. Batch size can be flexible and is based on the throughput rate and run time. With appropriate in-line testing, the process will stop if/when the product goes outside of the desired quality parameters, limiting potential loss. Consequently, continuous processing is being explored today by some pharmaceutical companies and research institutions.

##### Batch Size

The Food and Drug Administration 21CFR210.3 defines a lot as a batch, or a specific identified portion of a batch, having uniform character and quality within specified limits, or, in the case of a drug product produced by continuous process, it is a specific identified amount produced in a unit of time or quantity in a manner that assures its having uniform character and quality within specified limits (37).

Continuous processing equipment matches the raw material input with the processed product’s output. The unit will generate material at a controlled rate until the raw materials are exhausted or the process is stopped. Batch size is therefore variable and must be established by the manufacturer. Batch size is important in controlling potential liability (e.g., product recalls) and identifying the extent of release and stability testing required. Limiting a continuous process to a time range (e.g., 5 h or 5 days), introduction of new lots of raw materials (e.g., “batch” defined as the amount of product produced prior to a lot change in any raw material), or desired output (e.g., 500 kg) are all possible definitions that could be applied by a manufacturer.

##### Processors

Throughput with continuous systems is controlled by the equipment size and processing speed. Continuous processing has often meant large-scale equipment capable of producing metric tons of material a month. Recently, pharmaceutical equipment manufacturers have begun producing laboratory- or pilot-scale units for use in development and short-run production areas where flexibility is the desired goal. This equipment is often designed with quick-change product contact parts, allowing rapid production shifts. Smaller equipment also means the same units used for development may be adaptable to short-run production, thus eliminating scaling differences. These smaller units also allow more rapid prototyping with less material, reducing the cost and time of developing a design space.

##### Feeders

Maintaining appropriate control of raw material inputs is critical for continuous processing. An imbalance in material entering the unit will cause variation in processing conditions and output, potentially changing production uniformity. Feed rate control is usually accomplished by the use of highly accurate loss-in-weight feeders. Modern feeders can feed as little as 20 g/h and can operate with software capable of compensating for material powder flow issues inside the hopper.

##### Process Analytical Technology

PAT is critical to pharmaceutical continuous processing. Due to the need to modulate the process, in-line and real-time product testing with feedback loops to the controller is essential. PAT can also be used at other points in the process, with the potential to drastically reduce throughput times. For example, PAT tools such as near-IR could be applied to the raw material hoppers to provide within-drum and across-lot test data. This could be expanded to allow for online raw material release, eliminating or drastically reducing the wait time for chemical testing. PAT also increases the need for full electronic batch records to collate the sensor data streams and maintain correlation with processing parameters. The value of PAT application along the continuous process stream is obvious, monitoring product quality to meet the needs of continuous validation.

##### Quality by Design

QbD is an integral component of continuous processing, as is design of experiments. Continuous equipment allows for the effects of changes to processing speeds and materials to be rapidly assessed. Building the design space with continuous processing is more rapid due to the ability to change parameters and produce product more or less on the fly while consuming smaller amounts of material. With the low material holdup in most continuous systems, parameter or material changes can often be evaluated in a matter of minutes using minimal amounts of material. This statement will also be true of production systems, although the quantity of materials will increase with larger units.

##### Scale-Up

When appropriate-sized equipment is used in the developmental trials, process scale-up with continuous systems can be as simplistic as just running the unit for longer periods of time. This makes all of the design space data directly applicable to production operations with no need for extrapolations of the effects of larger equipment or different operating conditions. If larger equipment is necessary, more detailed validation can still be accomplished with less wasted material than under batch conditions.

##### Production

Production with continuous processing will resemble scale-up, with a further run time extension. All prior data are directly relevant and meaningful to the production operation. Changes to API or excipient can be evaluated directly on the production unit, but with significantly reduced risk compared to full-scale batch operations.

Continuous equipment can allow highly flexible operations with significantly reduced manufacturing floor space. This equipment will be much more automated than existing batch systems and could operate in near “lights out” conditions. Appropriate PAT development to allow in-line raw material testing and release would allow just-in-time raw material deliveries without the need to wait for QC testing. PAT applied to finished product coupled with complete electronic batch record information and automated data review could allow for real-time online release, reducing or eliminating the need for manual batch record review and release. With processors designed for rapid changeover, it is conceivable to operate in a lean environment and produce product to meet current consumer demand rather than to a predefined market forecast.

##### Systems

Vendors are beginning to produce systems capable of meeting the demands of continuous processing systems. Turnkey systems can be purchased for wet granulation and direct compression tablets, including operations through compression and coating. Equipment for semi-solids and some liquids have been developed, although usually as unique one-up systems. Computer systems capable of handling the wider data streams generated by modern PAT units, equipment inputs, and processing system control are more available and less expensive. These systems are capable of delivering the concept of continuous process validation. As PAT, computing systems, and production equipment improve, the potential for continuous processing equipment will expand.

##### Issues Affecting Implementation

While significant effort has been put into the development of continuous processing, commercial implementation is still limited. Gaining acceptance of PAT and full electronic batch record implementations has been challenging and is critical for the implementation of continuous systems. Some applications, e.g., lyophilization, are not normally applied in a continuous manner and present special equipment challenges. Existing capacity in batch facilities and equipment suggests that the cost of changeover to continuous systems is relatively high, resulting in pushback from current stakeholders. The lack of availability of these systems in developmental laboratories also reduces the likelihood of rapid implementation. The pharmaceutical industry is still waiting on the advent of breakthrough product or two to bring continuous processing to the forefront.

## Footnotes

1 If the formulations of all the strengths are not compositionally proportional, a biowaiver can be granted for the middle strengths based on acceptable BA or BE data for both the highest and the lowest strengths in the *in vitro* multimedia comparative dissolution profiles and *f*_2_ data.

2 If the formulations of all the strengths are not compositionally proportional, a biowaiver can be granted for the middle strength(s) based on acceptable BA or BE data as appropriate, for both the highest and the lowest strengths, and the *in vitro* multimedia dissolution comparison profiles and *f*_2_ data.

## Glossary of Scientific Terms Used in This Whitepaper

ANDA: Abbreviated new drug application
API: Active pharmaceutical ingredient
APR: Annual product review
BA: Bioavailability
BACPAC: Bulk active compound post-approval changes
BCS: Biopharmaceutics Classification System
BE: Bioequivalence
CANDA: Computer-aided NDA
CBE: Changes being effected
CMA: Critical manufacturing attribute
CMC: Chemistry, manufacturing and control
CMDh: Coordination Group for Mutual Recognition & Decentralized Procedures—Human (Canadian Ministry of Health)
CPP: Critical process parameter
CPV: Continued process verification
CQA: Critical quality attribute
DoE: Design of experiments
DS: Design space
DT: Disintegration time
EC: European Commission
EMEA: European Medicines Agency
FDA: Food & Drug Administration
FMEA: Failure mode effect analysis
FRC: Functionally related characteristics
HMI: Human–machine interface
HPLC: High-performance liquid chromatography
ICH: International Conference on Harmonization
IPC: In-process control
IR: Immediate release
IVIVC: *In vitro*–*in vivo* correlation
LOD: Loss on drying
MAPP: Manual of Policies & Procedures (FDA)
MR: Modified release or extended release
MSPC: Multivariate statistical process control
MVDA: Multivariate data analysis
NDA: New drug application
NIR: Near-infrared
NOC: Notice of change (Canadian Ministry of Health)
NOR: Normal operating range
OFAT: One factor at a time
PAR: Proven acceptable ranges
PAS: Prior approval supplement
PAT: Process analytical technology
PCA: Principal component analysis
PLS: Partial least squares
PNOC: Post-notice of compliance changes (Canadian Ministry of Health)
PSD: Particle size distribution
QbD: Quality by design
QRM: Quality risk management
QTPP: Quality target product profile
RTRT: Real-time release testing
SCADA: Supervisory Control and Data Acquisition
TPP: Target product profile
